# Recent Advances in Metal-Catalyzed Approaches for the Synthesis of Quinazoline Derivatives

**DOI:** 10.3390/molecules29102353

**Published:** 2024-05-16

**Authors:** Nitesh K. Nandwana, Om P. S. Patel, Manish K. Mehra, Anil Kumar, Joseph M. Salvino

**Affiliations:** 1 Medicinal Chemistry and Molecular and Cellular Oncogenesis (MCO) Program, The Wistar Institute, Philadelphia, PA 19104, USA; 2 Department of Technical Education, Government Polytechnic Naraini, Banda 210001, India; 3 Department of Chemistry, Birla Institute of Technology and Science, Pilani 333031, India

**Keywords:** quinazoline heterocycles, transition metal-catalyzed synthesis, C-H activation, cascade reactions, intramolecular dehydrative/dehydrogenative cyclizations

## Abstract

Quinazolines are an important class of heterocyclic compounds that have proven their significance, especially in the field of organic synthesis and medicinal chemistry because of their wide range of biological and pharmacological properties. Thus, numerous synthetic methods have been developed for the synthesis of quinazolines and their derivatives. This review article briefly outlines the new synthetic methods for compounds containing the quinazoline scaffold employing transition metal-catalyzed reactions.

## 1. Introduction

The development of novel and improved synthetic methods with or without the assistance of transition-metal catalysts for the synthesis and functionalization of a nitrogen-containing heterocyclic framework has always been a hot topic of research in synthetic organic chemistry due to their potential applications in the area of medicinal chemistry and material sciences [1,2,3,4,5,6].

**Figure 1:**
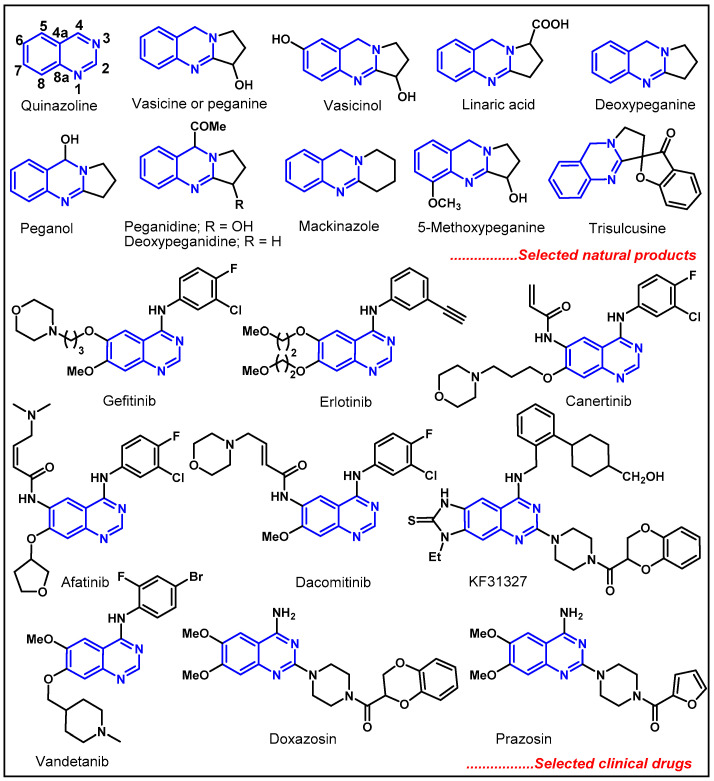
Molecular structures of selected natural products and clinical drugs containing quinazoline motif.

Nitrogen heterocycles may have significant potential in medicinal chemistry and drug discovery due to their ability to modify solubility, lipophilicity, and polarity, thereby changing the physicochemical properties and enhancing potency, selectivity, and metabolic stability [7,8]. Among all nitrogen heterocycles, quinazolines have attracted much attention from synthetic and medicinal chemists due to their ubiquity among various natural products, pharmaceuticals, functional organic materials, and agrochemicals [9,10,11,12,13,14]. As the main building block, the quinazoline core is found in several natural products isolated from plants, animals, and microorganisms [15,16]. The first natural quinazoline, vasicine, was isolated as a pure alkaloid by Hooper et al. in 1888. Later on, many natural products bearing the quinazoline skeleton were isolated from natural sources (Figure 1) [17,18,19]. More importantly, quinazoline derivatives exhibit a wide range of pharmacological activities, such as anti-malarial [20], anti-microbial [21], anti-inflammatory [22,23], anti-convulsant [24,25], anti-diabetic [26], anti-hypertensive [27], anti-cancer [28,29,30], anti-tumorous [31], anti-cholinesterase, dihydrofolate reductase inhibitory [32,33] and kinase inhibitory activities [34,35]. Additionally, quinazoline-based compounds serve as ligands for benzodiazepine and GABA receptors in the central nervous system, cellular phosphorylation inhibitors, and as DNA binding agents [36,37]. Quinazolines are the core structures in various clinical drugs, such as gefitinib, erlotinib, canertinib, vandetanib, afatinib, dacomitinib, prazosin, KF31327 and doxazosin, which are used for the treatment of various diseases (Figure 1) [38].

Owing to the importance of quinazolines, efficient routes for the synthesis of these compounds have received significant attention from synthetic communities over recent years [39,40]. Over the last decade, tremendous efforts have been made in utilizing various transition metal catalysts for the synthesis of simple and functionalized quinazolines. Transition metal-assisted methods have advantages over traditional methods in terms of reducing waste generation and the avoidance of pre-functionalized substrates, making the process more efficient and straightforward [41,42]. Another crucial improvement demonstrated by these catalytic reactions is the ability to employ simple substrates. This review describes a complete overview of the synthetic applications of transition metal-catalyzed reactions toward the preparation of substituted and polycyclic fused quinazolines from 2010 to 2022.

## 2. Classical Method 

**Scheme 1:**
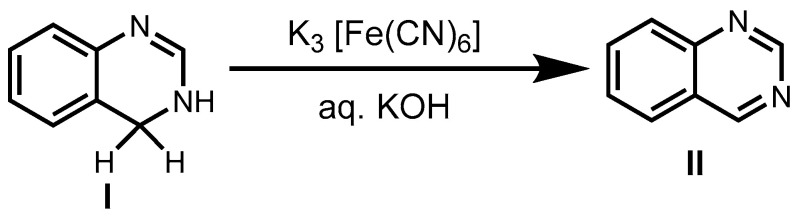
Gabriel method for quinazoline synthesis.

Due to the great importance of quinazoline derivatives across many fields, several synthetic methods have been developed to access quinazoline scaffolds. In 1903, Gabriel described the synthesis of quinazolines (**II**) via the oxidation of 3,4-dihydroquinazoline (**I**) (Scheme 1) [43]. 

**Scheme 2:**
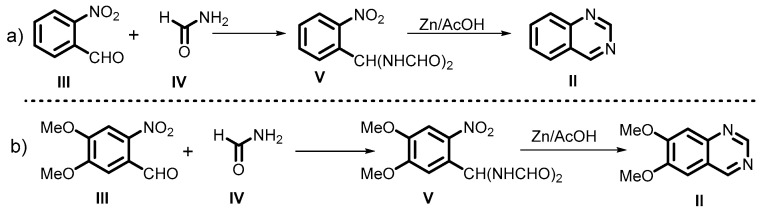
(**a**) Riedel method for quinazoline synthesis from *o*-nitrobenzaldehyde and amides; (**b**) Riedel method for quinazoline synthesis from using 6-nitro vertraldehyde and amides.

In 1905, Riedel described the synthesis of quinazolines (**II**) from *o*-nitrobenzaldehyde (**III**) and formamide (**IV**) in the presence of zinc and dilute acetic acid, leading to the production of corresponding quinazolines (**II**) with good yields (Scheme 2a). Later, Riedel modified the reaction conditions for an improvement in the reaction yield. This is one of the best methods for quinazoline synthesis, and has also been applied for the preparation of 6,7-dimethoxyquinazolines (**II**) by using 6-nitro vertraldehyde (**III**) and amide (**IV**) under similar reaction conditions (Scheme 2b) [44,45].

**Scheme 3:**
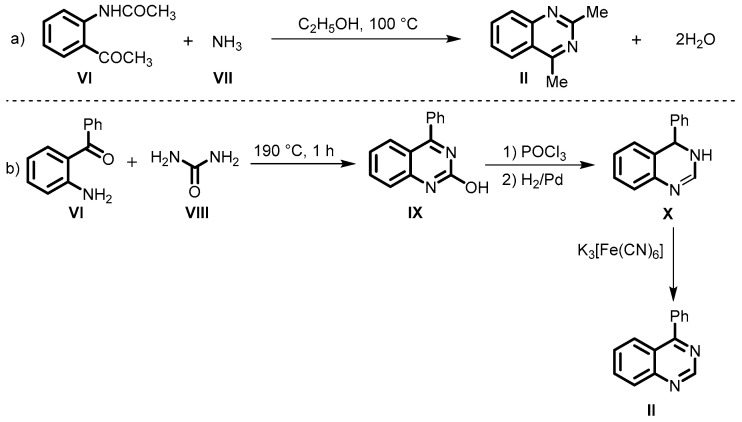
(**a**) Schofield method for the synthesis of 2,4-dimethyl quinazolines; (**b**) Schofield method for the synthesis of 4-phenyl quinazoline from *o*-amino benzophenone.

Schofield et al. described the synthesis of 2,4-dimethyl quinazolines (**II**) from substituted *o*-amino acetophenone (**VI**) and ammonia (**VII**) in ethanol at 100 °C (Scheme 3a) [46]. Subsequently, the same group synthesized 2-hydroxy-4-phenylquinazolines (**IX**) by means of the reaction of *o*-amino benzophenone (**VI**) with urea (**VIII**). The reaction of **IX** with POCl_3_ followed by reductive dehalogenation provided dihydroquinazoline (**X**), which on treatment with K_3_[Fe[CN]_6_ produced 4-phenyl quinazoline (**II**) (Scheme 3b).

The above-mentioned synthetic methods developed for quinazolines based on classical transformation suffer from some drawbacks, such as harsh reaction conditions, a stepwise procedure and a limited substrate scope. In the last decade, transition metal-catalyzed reactions have been employed to overcome these issues for the synthesis of quinazolines derivatives.

## 3. Manganese-Catalyzed Protocols

Manganese-catalyzed C-H activation reactions have recently been recognized as a powerful tool in organic synthesis due to the Earth-abundance of manganese and the inexpensive less toxic and eco-friendly nature of these reactions [47,48]. Manganese is the twelfth most abundant element in the Earth’s crust and the third most abundant transition metal. The growing need for less toxic and less expensive methods prompted researchers to design and synthesize a quinazoline scaffold using Mn-catalyzed C-H functionalization reactions.

**Scheme 4:**
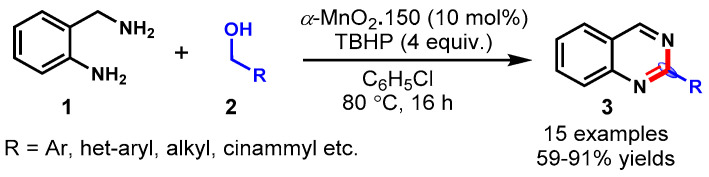
Synthesis of quinazolines from 2−amino−benzylamines and alcohols.

In 2015, Wang and his group developed a robust and reusable method for the *α*-MnO_2_-catalyzed synthesis of quinazolines (**3**) from 2-amino-benzylamines (**1**) with alcohols (**2**) in the presence of TBHP as an oxidant in chloro-benezene solvent (Scheme 4) [49]. Various alcohols, including aromatic alcohols, heterocyclic alcohols and aliphatic alcohols, reacted with 2-amino-benzylamines under optimized conditions and delivered the desired products with 59–91% yields. A radical-mediated mechanism was deduced for this transformation based on the control experiments using a radical quencher, i.e., TEMPO (2,2,6,6-tetramethylpiperidin-1-yl)oxy) and BHT (butylated hydroxy toluene), in the reaction.

**Scheme 5:**
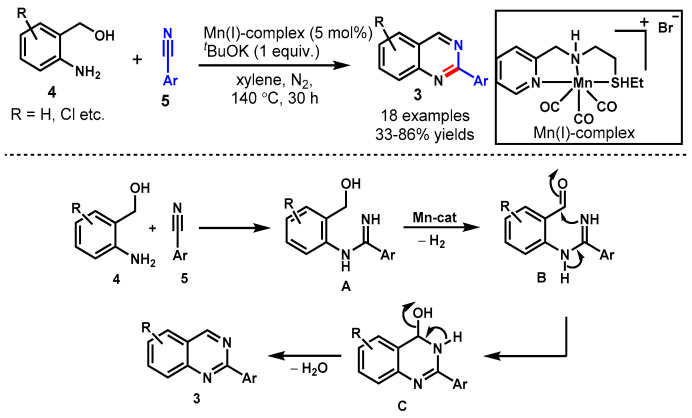
Synthesis of quinazolines from 2−aminobenzylalcohols and nitriles.

In 2019, Das et al. described an acceptorless dehydrogenative annulation strategy for the synthesis of quinazolines (**3**) from 2-amino-benzylalcohols (**4**) and nitriles (**5**) in the presence of the phosphine-free Earth-abundant well-defined Mn(I) pincer complex (Scheme 5) [50]. A broad range of EDGs and EWGs on both the aryl nitriles and 2-amino-benzylalcohols were well tolerated and delivered the desired products in good yields under optimal conditions. In contrast, the reaction of an aliphatic nitrile, i.e., valeronitrile, produced a complex mixture and the desired product was isolated with only a 10% isolated yield. The present transformations eliminated H_2_O and H_2_ gas as side products. The evolved H_2_ gas was used to transform styrene to ethylbenzene through a Pd/C-catalyzed hydrogenation reaction.

**Scheme 6:**
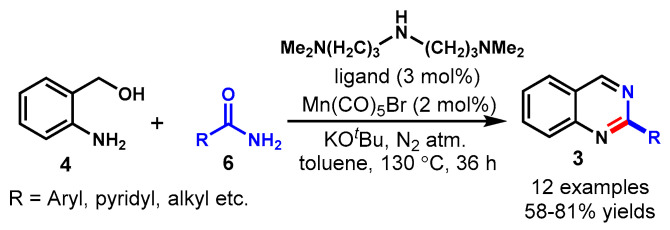
Synthesis of quinazolines from 2–aminobenzyl alcohol and primary amides.

In 2020, Balaraman and his group developed a Mn(I)-catalyzed acceptorless dehydrogenative coupling strategy of 2-amino-benzylalcohol (**4**) with primary amides (**6**) for the synthesis of 2-substituted quinazolines (**3**) in toluene at 130 °C (Scheme 6) [51]. A variety of aryl and alkyl amides reacted well with 2-aminobenzyl alcohol to deliver the respective products in 58–81% yields under the optimized reaction conditions. Limitations included trans-cinnamamide and 2-F-/2-NO_2_-substituted benzamides, since the developed method failed to produce the desired products for these substrates when they were subjected to a similar set of reaction conditions. The Mn(I)-catalyst and KO*^t^*Bu base displayed an important role in this transformation by facilitating the dehydrogenative and condensation steps, respectively.

**Scheme 7:**
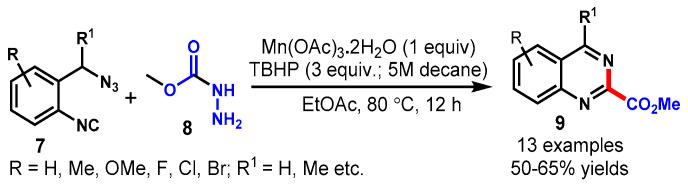
Synthesis of quinazolines from 2–(azidomethyl)–phenyl isocyanides and methyl carbazate.

Also in 2020, Begum and co-workers reported a Mn/TBHP-mediated oxidative radical cyclization of 2-(azidomethyl)phenyl isocyanides (**7**) and methyl carbazate (**8**) for the synthesis of quinazoline-2-carboxylates (**9**) in an eco-friendly solvent, i.e., ethyl acetate (Scheme 7) [52]. Different functional groups on 2-(azidomethyl)phenyl isocyanides were well tolerated under optimized conditions. The radical intermediate (**•**CO_2_Me) was generated via the oxidative decomposition of methyl carbazate into the reaction, as confirmed by NMR and LCMS analysis, which further reacted with 2-(azidomethyl)phenyl isocyanide to provide the respective product. In general, this protocol represented a novel, reliable, straight-forward, atom-efficient method for the synthesis of methyl quinazoline-2-carboxylates in good yields.

## 4. Iron-Catalyzed Protocols

Iron is the most abundant transition metal present in the Earth’s crust. Iron’s ready availability, low toxicity, low cost, variable oxidation states, and ligand binding abilities make iron catalysis a promising area for chemical synthesis. Iron catalysis has become a rapidly growing area of research in various chemical transformations, such as C-H activation reactions [53], cycloaddition reactions [54], and substitution reactions [55]. Iron catalysis has also been used for the design and synthesis of quinazoline derivatives.

**Scheme 8:**
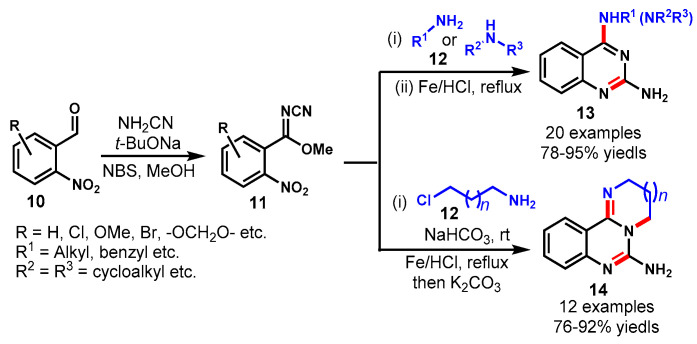
Synthesis of 2,4-diaminoquinazolines and tricyclic quinazolines.

In 2012, Yin et al. developed an efficacious route for the synthesis of 2,4-diaminoquinazolines (**13**) and tricyclic quinazolines (**14**) via cascade reductive cyclization of methyl *N*-cyano-2-nitrobenzimidates (**11**) in the presence of an Fe/HCl system (Scheme 8) [56]. The key substrate, i.e., methyl *N*-cyano-2-nitrobenzimidates, was produced in a quantitative yield via the cyanoimidation of 2-nitrobenzaldehyde (**10**) with NH_2_CN, which further reacts with different primary amines (**12**) substituted with aliphatic chains or rings to produce the respective diamino quinazolines products in 78–95% yields. Moreover, the tricyclic quinazolines, i.e., 2,3-dihydroimidazo[1,2-*c*]quinazolin-5-amines (**14**), are synthesized in good yields by treating various methyl *N*-cyano-2-nitrobenzimidate with 2-chloro-ethanamine/3-chloropropan-1-amine under the developed reaction conditions. The formation of two heterocycles in a one-pot procedure is the most advantageous aspect of this methodology. Notably, the cascade ring-opening/ring-closing of tricyclic quinazolines was demonstrated to produce quinazolinones bearing a cyclic guanidine motif.

**Scheme 9:**
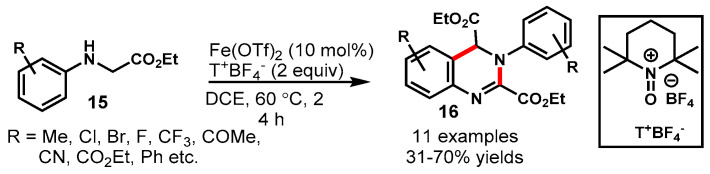
Synthesis of diethyl 3–aryl–3,4–dihydroquinazoline–2,4–dicarboxylates.

In 2013, Rohlmann et al. explored the oxidative tandem synthesis of dihydroquinazolines (**16**) from *N*-alkylanilines (**15**) in presence of Fe(OTf)_2_ as a catalyst and TEMPO oxoammonium salt as a mild and nontoxic oxidant (Scheme 9) [57]. The developed method tolerated a wide range of electron-deficient functional groups on *N*-alkylanilines to provide the desired products in good yields. On the other hand, the mono-substituted *o*-Me and *o*- and *m*-OMe groups bearing substrates produced a complex reaction mixture in which the respective products were not identified. This might be due to the relatively higher reactivity of electron-rich anilines toward both oxidation and aromatic nucleophilic substitution, as deduced by the authors. In this transformation, an iminium species is a key intermediate produced from the initial α-oxidation of *N*-alkylanilines by TEMPO salt.

**Scheme 10:**
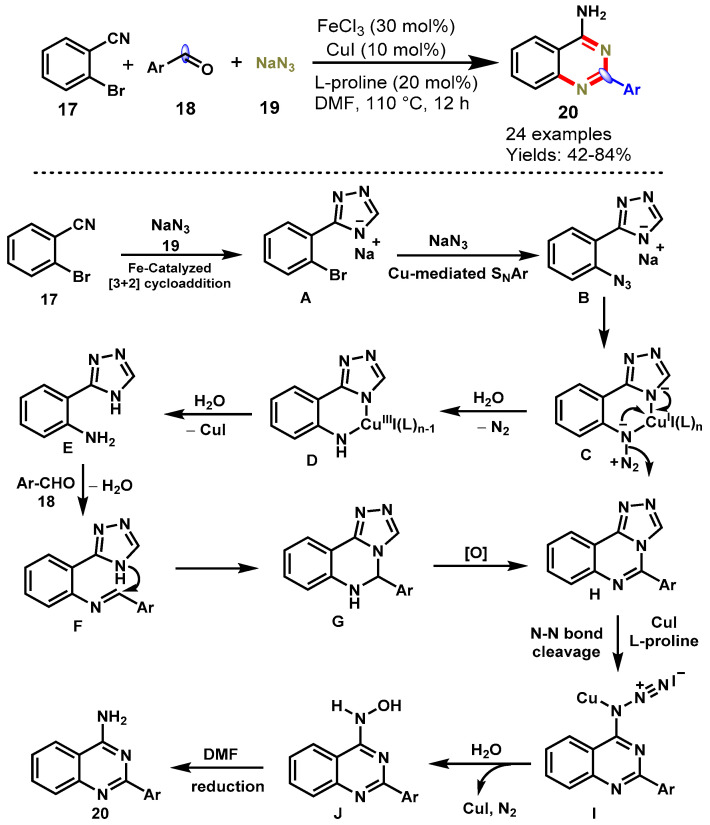
Synthesis of 2-phenylquinazolin-4-amines via a Fe/Cu relay-catalyzed domino strategy.

In 2015, Wu and co-workers described a highly efficient Fe/Cu relay-catalyzed domino protocol for the synthesis of 2-phenylquinazolin-4-amines (**20**) from commercially available ortho-halogenated benzonitriles (**17**), aldehydes (**18**), and sodium azide (**19**) (Scheme 10) [58]. Mechanistically, the reaction proceeded through iron-mediated [3 + 2] cycloaddition, copper-catalyzed S_N_Ar, reduction, cyclization, oxidation, and denitrogenation sequences. A variety of aromatic and heteroaromatic aldehydes bearing different substituents like ERGs (Me, OMe, OEt) and EWGs (Cl, Br, NO_2_) were employed smoothly with ortho-halogenated benzonitriles under optimized conditions to provide corresponding products in moderate to good yields (42–84%). In addition, this approach was successfully applied for the synthesis of 1-(2-methoxyphenyl)-3-(2-(pyridin-3-yl)quinazolin-4-yl)urea (IV) under optimal conditions, which is a potent human adenosine A3 receptor antagonist.

**Scheme 11:**
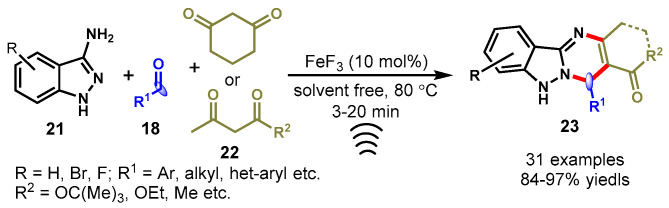
Synthesis of functionalized indazolo[3,2-*b*]quinazoline derivatives.

In 2016, Shinde et al. demonstrated an FeF_3_-catalyzed three-component approach for the synthesis of functionalized tetrahydroindazolo[3,2-*b*]quinazolines (**23**) using 1*H*-indazole-3-amines (**21**), aldehydes (**18**), and cyclic and acyclic 1,3-diketones (**22**) under sonication in solvent-free conditions (Scheme 11) [59]. The developed method showed a broad range of substrate scopes and successfully delivered the respective products in excellent yields. There are many advantageous features of this methodology as highlighted by the authors, such as a shorter reaction time, a low cost, an easy work-up procedure, and the bypass of using solvent and column chromatography. Moreover, FeF_3_ displayed high catalytic activity and it can be readily recovered and reused further without a significant loss in the yields of the respective products.

**Scheme 12:**
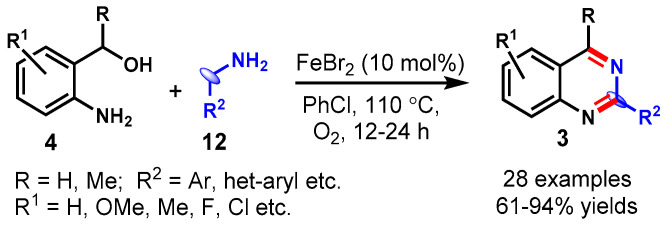
Synthesis of quinazolines from 2-aminobenzyl alcohols with benzylamines.

In 2017, Gopalaiah and co-workers realized the FeBr_2_-catalyzed cascade synthesis of C-2 substituted quinazolines from 2-aminobenzyl alcohols (**4**) with benzylamines (**12**) under aerobic oxidative conditions (Scheme 12) [60]. The developed method tolerated a wide range of substrates and functional groups to produce the respective 2-arylated/heteroarylated quinazolines in good to excellent yields. Disappointingly, aliphatic amines were found to be nonreactive to this transformation under the optimal reaction conditions. The present method was scaled up to a 20 mmol scale, yielding 2-phenylquinazoline in an 86% yield without a significant loss in reaction efficacy. Notably, 7-chloro-2-phenylquinazoline, an IGF-IR (Insulin-like Growth Factor-I Receptor) enzyme inhibitor, was also synthesized using the developed reaction conditions.

**Scheme 13:**
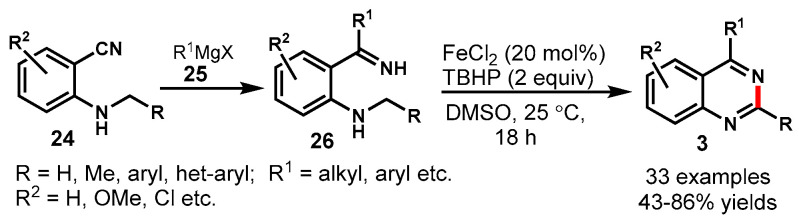
Synthesis of 2,4-disubstituted quinazolines from 2-alkylamino N-H ketimines.

In 2018, Chen and co-workers demonstrated the synthesis of quinazolines from 2-alkylamino N-H ketimines (**26**) through FeCl_2_-catalyzed sp^3^ C-H oxidation and intramolecular C-N bond formation using TBHP as the terminal oxidant (Scheme 13) [61]. A variety of 2-alkylamino N-H ketimines (**26**) were successfully obtained from the 2-alkylamino benzonitriles (**24**) with the addition of Grignard or organolithium reagents (**25**). The developed protocol smoothly furnished the respective C-2 alkylated/arylated/-heteroarylated quinazolines in good to excellent yields. Moreover, using the developed method, a muscle relaxer drug, i.e., quazodine (4-ethyl-6,7-dime-thoxy quinazoline), was synthesized in a 72% yield. The developed approach worked well for the scaled-up synthesis of quinazoline.

**Scheme 14:**
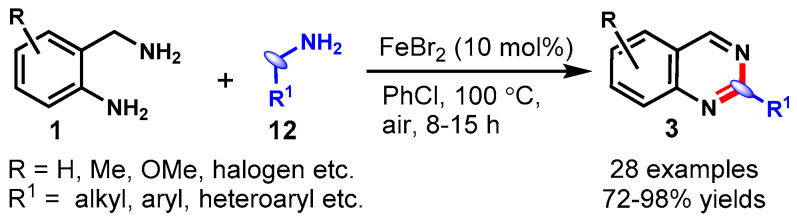
Synthesis of quinazolines from 2-aminobenzyl-amines and amines.

In 2019, Gopalaiah and his research group developed a method for the FeBr_2_-catalyzed synthesis of quinazolines from 2-aminobenzylamines (**1**) and amines (**12**) in chlorobenzene as a solvent at 100 °C under aerobic conditions (Scheme 14) [62]. Various benzylamines, heteroarylmethanamines, and alkylamines reacted smoothly with electronically variable 2-aminobenzylamines under optimized conditions, yielding the respective products in good to excellent yields. The reaction of phenyl glycine as a benzylamine surrogate successfully delivered the desired quinazoline product in a 57% yield. Mechanistically, this oxidative condensation pathway proceeded through imine generation through the self-coupling of benzylamine, trans-imination, and intramolecular C-N coupling, followed by aromatization.

## 5. Cobalt-Catalyzed Protocols

Cobalt-catalyzed C–H activation and C-N bond formation have received major advancements in the past couple of decades [63,64]. Cobalt is one of the most promising first-row transition metals for catalysis. Cobalt is a low-cost and less toxic metal with variable oxidation states and high chemoselectivity. Therefore, it has a wide range of applications in the chemical synthesis of natural products, pharmaceuticals, and other organic molecules.

**Scheme 15:**
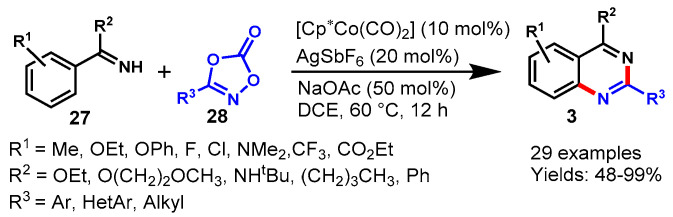
Cobalt-catalyzed [4 + 2] cycloaddition of imines with dioxazolones.

In 2016, Glorius and team achieved the Cp*Co(III)-catalyzed [4 + 2] cycloaddition reaction of imines (**27**) with dioxazolone (**28**) using AgSbF_6_ as an oxidant and sodium acetate as a base in DCE (Scheme 15) [65]. The mechanism of the reaction involved cobalt-catalyzed tandem direct C-H amidation followed by intramolecular cyclization. Diversely substituted arylimidates reacted smoothly with dioxazolones bearing various functional groups at different positions of the aryl ring under standard reaction conditions to provide the corresponding products in good to excellent yields (48–99%). Moreover, the developed protocol has been successfully demonstrated for the synthesis of biologically active compounds such as Bacillus cereus, antimetabolite drug raltitrexed, and ICI 198583. Notably, the cobalt complex (Cp*Co(III)) displayed significantly higher activity than Cp*Ir(III) and Cp*Rh(III) complexes. This might be possible due to the strong Lewis acidity and the high sensitivity to steric hindrance of Cp*Co(III) catalyst.

**Scheme 16:**
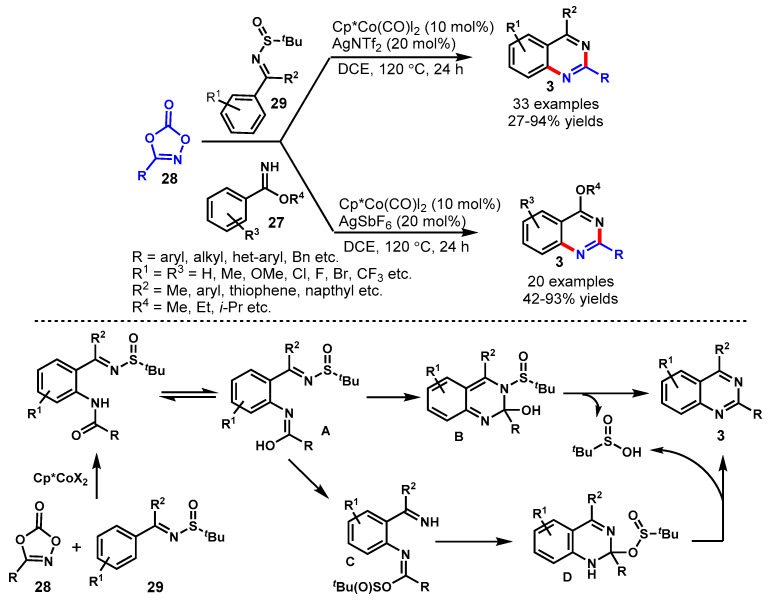
Synthesis of 4-aryl-/alkoxy-quinazolines from dioxazolones.

In 2016, Li and co-workers established Co(III)-catalyzed C-H bond activation approaches for the synthesis of two different types of quinazolines from *N*-sulfinylimines (**29**) and benzimidates (**27**) using dioxazolones (**28**) as a nitrile surrogate in DCE solvent (Scheme 16) [66]. A broad range of *N*-sulfinylimine and benzimidate substrates decorated with EDGs and EWGs treated with aryl-/alkyl-dioxazolones under different reaction conditions and two different series of desired products (4-arylquinazolines and 4-alkoxyquinazolines) were synthesized in good to excellent yields. It is noteworthy that alkyl dioxazolones have shown relatively poor reactivity with *N*-sulfinylimines as compared to the aryldioxazolones. On the other hand, the reactivity of alkyl dioxazolones was equally efficacious with benzimidates. The 4-ethoxy-quinazoline was further transformed into the corresponding quinazolinone under acidic hydrolysis.

**Scheme 17:**
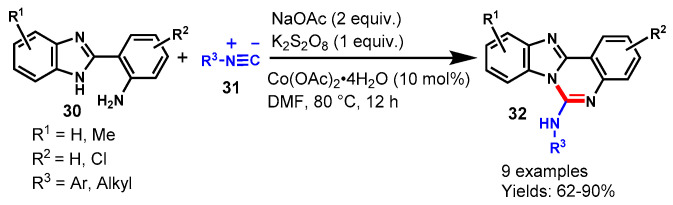
Cobalt-catalyzed isocyanide insertion reaction.

In 2016, Ahmadi et al. described a cobalt-catalyzed isocyanide insertion cyclization reaction for the synthesis of benzoimidazoquinazoline amines (**32**) via the reaction of isocyanides (**31**) and benzo[*d*]-imidazol-anilines using (**30**) K_2_S_2_O_8_ as an oxidant and sodium acetate as a base in DMF (Scheme 17) [67]. Various substituted isocyanides and benzo[*d*]-imidazol-anilines participated well under optimized conditions and delivered the desired products in moderate to excellent yields (62–90%). In addition, the developed methodology has been successfully applied to the synthesis of tetrazoloquinazolin-5-amine and quinazolin-4(3*H*)-one under similar reaction conditions.

**Scheme 18:**
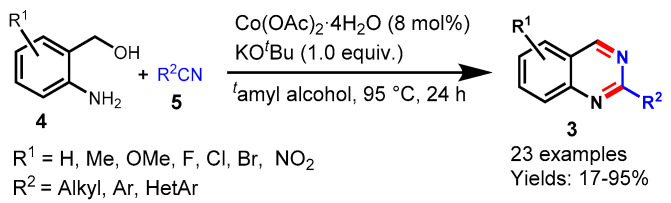
Cobalt-catalyzed quinazoline synthesis from 2-aminoaryl alcohols and nitriles.

In 2022, Hao et al. described the dehydrogenative synthesis of quinazoline via the ligand-free cobalt-catalyzed annulation of 2-aminoaryl alcohols (**4**) with nitriles (**5**) under mild reaction conditions. The authors examined the effects of various substituents on benzonitrile and found that aryl nitrile containing methyl and methoxy underwent smoother dehydrogenative annulation than aryl nitrile containing NO_2_ groups. Moreover, heteroaromatic and aliphatic nitriles were less reactive for annulations reactions. 2-aminoaryl alcohols bearing ERGs and EWGs were well tolerated under standard reaction conditions and provided the target products in a good yield (48–77%) (Scheme 18) [68].

## 6. Copper-Catalyzed Protocols

Copper-catalyzed C-H activation reactions have emerged as the most convenient and efficient method for the construction of complex heterocycles from the simple starting material [69]. Over the past few years, copper salts have shown their significant importance in organic transformation because of their low toxicity and inexpensive nature. Recently, the development of novel methodologies through copper-catalyzed C-N coupling reactions have received considerable attention due to their important physiological and biological activities. In particular, copper-catalyzed methodologies relating to the synthesis of quinazoline have been widely explored by researchers.

**Scheme 19:**
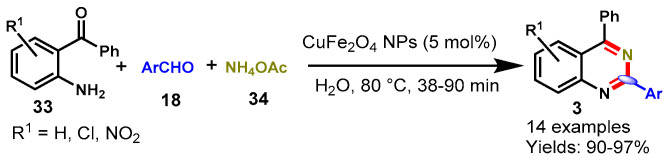
CuFe_2_O_4_ nanoparticles-catalyzed synthesis of 2-aryl quinazolines.

In 2014, Farhang’s group reported an efficient one-pot tandem cyclization between 2-amino benzophenones (**33**), aryl aldehydes (**18**), and NH_4_OAc (**34**) using magnetically separable and reusable CuFe_2_O_4_ nanoparticles in aqueous media (Scheme 19) [70]. The CuFe_2_O_4_ nanoparticles were prepared via the thermal decomposition of Cu(NO_3_)_2_ and Fe(NO_3_)_3_ in water in the presence of sodium hydroxide. Substituted 2-aminobenzophenones and various aromatic aldehydes reacted efficiently under optimized conditions and afforded the desired products in excellent yields (90–97%). In addition, the catalytic activity of CuFe_2_O_4_ nanoparticles was evaluated in aqueous media, displaying its applicability as a green, reusable and promising catalyst in organic synthesis.

**Scheme 20:**
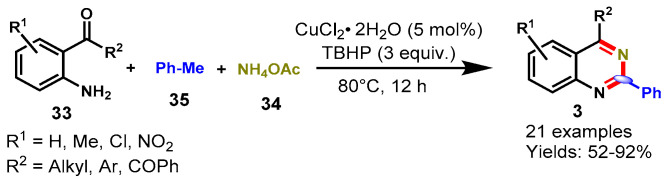
Synthesis of quinazolines from 2-aminobenzo-ketones and toluene.

In 2015, Wang and team achieved direct access to functionalized quinazolines through the reaction of 2-aminoarylketones (**33**), methylarenes (**35**) and NH_4_OAc (**34**) via copper-catalyzed dual oxidative benzylic C-H aminations of methylarenes using TBHP as an oxidant (Scheme 20) [71]. The developed approach was successfully employed for the synthesis of quinazolines bearing ERG and EDG functional groups in good to excellent yields (52–92%). Mechanistically, the reaction proceeded through the oxidative amination of the benzylic C-H bond of methyl arenes with ammonia and 2-aminoarylketones followed by intramolecular cyclization. Moreover, the kinetic isotope effect (KIE) described that the C-H bond cleavage is the rate-determining step in this methodology.

**Scheme 21:**
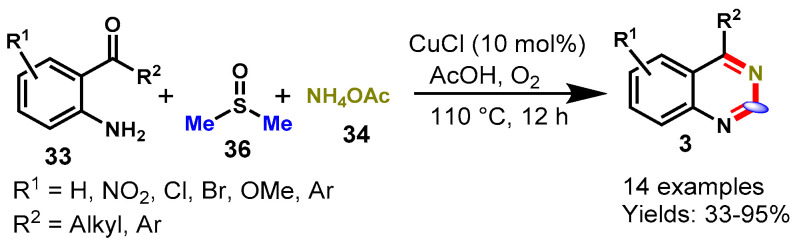
Synthesis of quinazolines from 2-amino-benzoketone and DMSO.

In 2016, Ma’s group reported a CuCl-catalyzed three-component oxidative amination reaction from ortho-carbonyl anilines (**33**), with NH_4_OAc (**34**) as a nitrogen source and DMSO (**36**) as a carbon source, in acetic acid under an oxygen atmosphere for quinazoline synthesis (Scheme 21) [72]. The scope of this methodology was explored by varying different functional groups like ERGs and EWGs on the aryl ring of ortho-carbonyl anilines to provide desired products in moderate to excellent yields (33–95%). Furthermore, authors also explored different carbon sources such as DMSO, DMF, DMA, *N*-methylacetamide, TMEDA, DMEDA, *N*-methyl-2-pyrrolidone with ortho-carbonyl anilines under standard reaction conditions, but among them, DMSO was found to be a more appropriate carbon synthon for this method. Moreover, the DMSO-*d*6 experiment was conducted to prove that the carbon atom in the product derived from DMSO. The radical trapping experiment was performed by using TEMPO, which revealed that the reaction proceeded through a radical pathway.

**Scheme 22:**
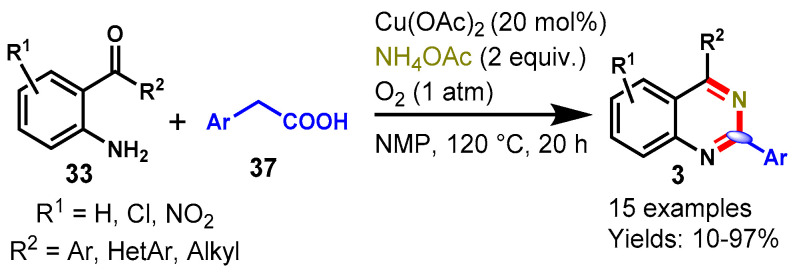
Synthesis of quinazolines from 2-aminobenzo-ketones and aryl acetic acids.

In 2016, Liu and colleagues demonstrated Cu(OAc)_2_-catalyzed aerobic oxidative decarboxylative amination of aryl acetic acids (**37**) with 2-aminobenzoketones (**33**) and ammonium acetate under an oxygen atmosphere in NMP at 120 °C (Scheme 22) [73]. Different functional group decorated 2-amino-benzoketones including aromatic, hetero-aromatic, and aliphatic substituents on aryl ring treated well with aryl acetic acid under standard reaction conditions and provided the corresponding products in 10–97% yields. Radical trapping experiments were conducted by using TEMPO, which revealed that the reaction might proceed via a radical pathway. There are some advantageous features of this approach that make it an efficient method, such as molecular oxygen being as the sole oxidant, its operational simplicity, having H_2_O and CO_2_ as wastes, and multiple C-N bond formation via C-H and C-C bond cleavage.

**Scheme 23:**
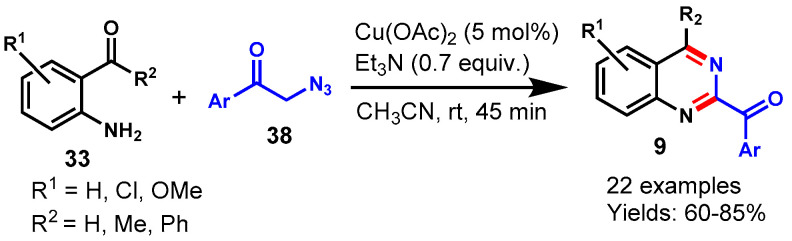
Synthesis of quinazolines from 2-aminobenzo-phenones and phenacyl azides.

In 2017, Sastry et al. described an excellent Cu(OAc)_2_-catalyzed method between 2-amino benzaldehyde/-ketone (**33**) and phenacylazides (**38**) for functionalized quinazolines (**9**) using triethylamine as base in acetonitrile at room temperature (Scheme 23) [74]. 2-Aminobenzaldehyde/ketone bearing ERGs (Me, OMe) and EWG (Cl) reacted smoothly with various substituted phenacylazides under optimized conditions and delivered the respective quinazolines in 60–85% yields. The mechanism of the reaction proceeded through involvement of phenacylazides for the generation of imine precursors for transimination with ortho-carbonyl anilines followed by condensation leading to respective aroyl-substituted quinazoline derivatives.

**Scheme 24:**
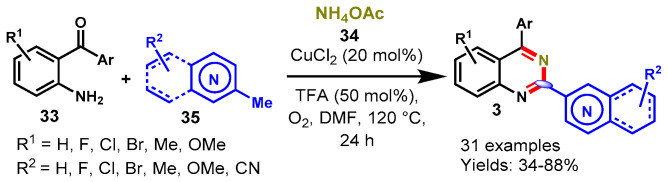
Synthesis of quinazolines from 2-aminobenzophenones and methylazaarenes.

In 2019, Liang et al. explored a CuCl_2_-catalyzed one-pot three-component aerobic oxidative cyclization approach for the synthesis of quinazolines via the amination of C(sp^3^)-H bonds of methylazaarenes (**35**) in the presence of ammonium acetate (**34**) and TFA in DMF at 120 °C under an oxygen atmosphere (Scheme 24) [75]. Various ERGs and EWGs at different positions of the aryl ring of 2-aminophenylketone (**33**) reacted smoothly with methylazaarenes under standard reaction conditions and delivered the corresponding products in moderate to very good yields. Moreover, methylazaarens containing multiple heteroatoms such as 2-methylpyrazine, 4-methylpyrimidine, 2-methyl-thiazole, and 2-methylbenzothiazole also worked well, affording the corresponding azaaryl-substituted quinazolines in 34–69% yields.

**Scheme 25:**
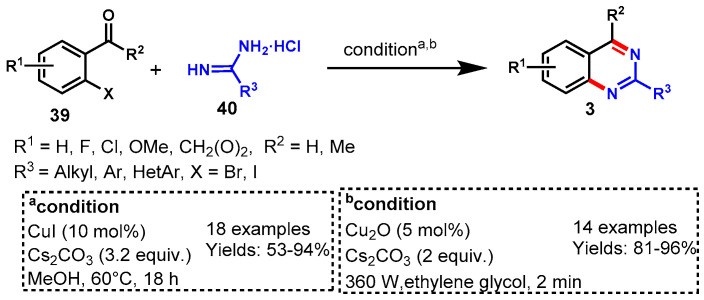
(**a**) Synthesis of quinazolines from ortho-iodobenzaldehydes and amidine hydrochlorides; (**b**) Ultrasound-assisted synthesis of quinazolines from 2-bromobenzaldehydes and amidine.

In 2010, Morrow’s group developed a CuI-catalyzed Ullmann condensation for the synthesis of highly functionalized quinazolines from the reaction of ortho-iodobenzaldehydes (**39**) with amidine hydrochlorides (**40**) using Cs_2_CO_3_ as the base in methanol at 60 °C. The substituted 2-iodobenzaldehye reacted smoothly with various functional group-containing benzamidine hydrochlorides such as methoxy, fluoro, trifluoromethyl, and heterobenzamidine hydrochlorides to produce the respective quinazolines in 53–94% yields (Scheme 25a) [76]. Dramatically, authors observed lower yields when ortho-bromobenzaldehyde was used as the substrate. To further improve the reaction yields, the reaction temperature was increased from 60 °C to 100 °C.

In 2017, Bhanage and his colleagues improved the reaction conditions by using facile and green ultrasound-assisted Cu_2_O nanocubes at room temperature. The authors demonstrated the synthesis of quinazolines from 2-bromobenzaldehydes (**39**) with amidines (**40**) under ligand-free conditions by using Cu_2_O nanocubes as a heterogeneous nanocatalyst. Various substituted 2-bromobenzaldehydes reacted smoothly with aromatic and aliphatic amidines under stabilized reaction conditions and delivered the respective products in very good to excellent yields (81–96%). The developed protocol has some advantageous features which make it a good approach for the synthesis of functionalized quinazolines, such as a shorter reaction time, a green solvent, catalyst recyclability, and high stability (Scheme 25b) [77].

**Scheme 26:**
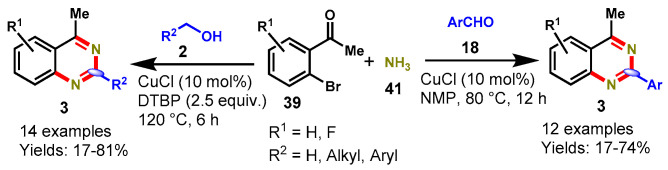
Synthesis of quinazolines from ortho-bromoaromatic ketones and aldehydes or alcohols.

In 2012, Ju et al. explained a CuCl-catalyzed one-pot multi-component approach for the synthesis of quinazolines (**3**) via the reaction of ortho-bromoaromaticketones (**39**) with aldehydes (**18**) or alcohols (**2**) using ammonia water (**41**) as the nitrogen source and air or DTBP as an oxidant (Scheme 26) [78]. A variety of aldehydes containing EDGs and EWGs reacted well with ortho-bromoaromatic ketones to produce the desired quinazoline derivatives in poor to good yields (17–74%). In addition, 2-acetyl-3-bromothiophene also worked well under the stabilized conditions to afford the respective product in 43% yield. Unfortunately, aliphatic aldehydes and 4-nitrobenzaldehyde displayed low reactivity and selectivity toward quinazolines synthesis. Further, the authors improved the reaction condition to avoid aldol condensation in the case of aliphatic aldehydes and conducted an oxidation process of primary alcohols to aldehydes using DTBP as an oxidant. The developed protocol worked well with aromatic as well as aliphatic alcohol to produce the respective quinazolines in 17–81% yields. Mechanistically, the reaction proceeded through amination and condensation, followed by the oxidation process.

**Scheme 27:**
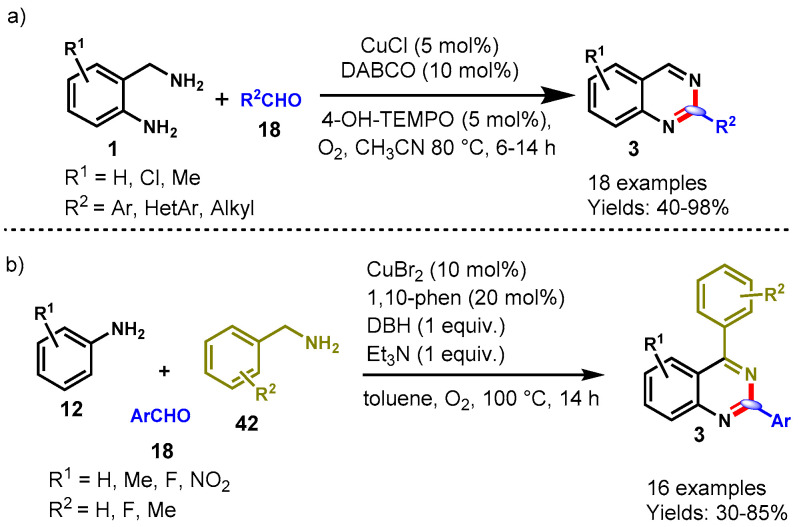
(**a**) Copper-catalyzed synthesis of quinazolines from aldehyde and benzylamine; (**b**) One-pot three component annulation approach for the synthesis of quinazoline derivatives frombenzaldehydes, benzyl amines and anilines.

In 2011, Yu and colleagues described a one-pot reaction of 2-aminobenzylamines (**1**) with aryl aldehydes (**18**) for the synthesis of quinazolines by employing CuCl/DABCO/4-HO-TEMPO as the catalysts and oxygen as an oxidant in CH_3_CN at 80 °C (Scheme 27a) [79]. Various substituted aldehydes (aromatic, heteroaromatic and aliphatic) were treated with a range of substituted 2-aminobenzylamines under standard reaction conditions, providing the substituted quinazolines (**3**) in moderate to excellent yields (40–98%). However, the authors observed lower yields in the case of aliphatic aldehyde and cinnamaldehyde than for aromatic aldehydes. Moreover, heterocyclic aldehydes such as 3-picolylaldehyde and 2-furyl aldehyde also worked well under optimized conditions. The intermolecular kinetic isotopic effects were also analyzed to establish the mechanistic pathway. This was the first method for the synthesis of quinazolines through oxidative dehydrogenation by using the CuCl/DABCO/4-HO-TEMPO catalytic system.

Recently, in 2021, Liu et al. demonstrated a one-pot three component annulation approach for the synthesis of quinazoline derivatives through the reactions of diversely substituted benzaldehyde (**18**), benzyl amine (**42**) and anilines (**12**) (Scheme 27b) [80]. In addition, these authors described the electronic effect of various substituents on the benzene ring and observed that electron-donating substituents were well tolerated in comparison to substrates containing electron-withdrawing groups. Furthermore, the authors also described that F- and NO_2_-containing aniline failed to provide the desired product under optimal conditions.

**Scheme 28:**
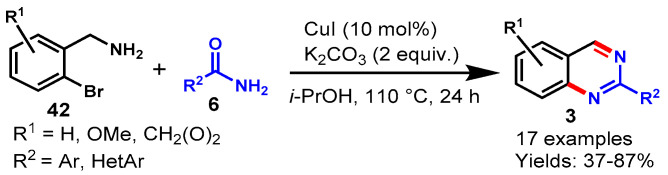
Synthesis of quinazolines from (2-bromo-phenyl)-methylamines and amides.

In 2010, Fu and co-workers reported a CuI-catalyzed tandem approach for the synthesis of quinazoline derivatives from readily available (2-bromophenyl)-methylamine (**42**) and amide (**6**) derivatives using K_2_CO_3_ as a base and 2-propanol as the solvent under air at 110 °C. (Scheme 28) [81]. The scope of the reaction was explored by using a range of electron-rich (Me, OMe) and electron-deficient (F, NO_2_) substituents on amide with (2-bromophenyl)-methylamines, and all the derivatives reacted well to provide the respective products in 37–87% yields. Moreover, (2-bromophenyl)methylamines containing EDGs showed lower reactivity than ERGs. However, aliphatic amides could not produce the respective products under optimized conditions. Mechanistically, the reaction proceeded through copper-catalyzed sequential Ullmann-type coupling followed by intramolecular nucleophilic addition and aromatization under aerobic conditions.

**Scheme 29:**
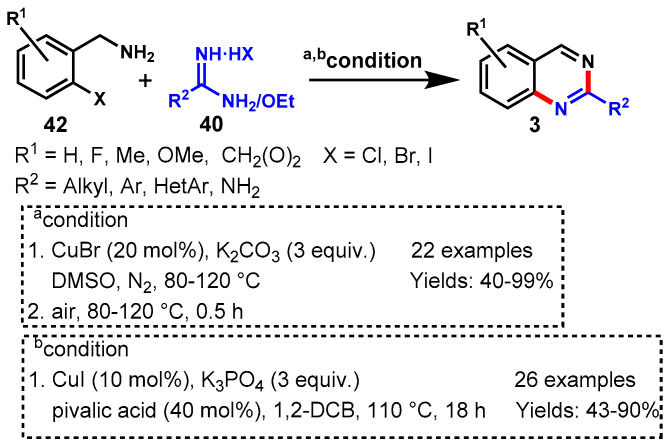
(**a**) Synthesis of quinazolines from (2-halophenyl)methylamines and amidines; (**b**) CuI-catalyzed tandem reaction for the synthesis of functionalized quinazolines.

In 2013, Cheng’s group described the CuBr-catalyzed one-pot tandem approach for the synthesis of substituted quinazolines from the reaction of 1-(2-halophenyl)methanamines (**42**) and amidines (**40**) using K_2_CO_3_ as a base in DMSO under a nitrogen atmosphere (Scheme 29a) [82]. Mechanistically, the reaction proceeded via sequential intermolecular *N*-arylation and intramolecular nucleophilic substitution followed by aerobic oxidation. Different functional groups decorating 1-(2-halophenyl)methanamines reacted efficiently with guanidine hydrochloride under standard reaction conditions to provide substituted quinazolines in moderate to excellent yields (40–99%). Notably, aromatic amidines containing electron-withdrawing groups displayed lower reactivity than those containing electron-donating groups.

Later on, Omar et al. developed a CuI-catalyzed tandem reaction for the synthesis of functionalized quinazolines through the reaction of 1-(2-bromophenyl)-methanamines and amidines using K_3_PO_4_ as the base, pivalic acid as the additive, and oxygen as the oxidant in 1,2-DCB at 110 °C (Scheme 29b) [83]. Various substituted benzamidine salts, including aromatic, heteroaromatic, and aliphatic, reacted well with different functional groups containing 1-(2-bromo-phenyl)methanamine under optimized conditions to deliver the respective products in 43–90% yields. Furthermore, authors successfully extended their work for the synthesis of quinazolines by using ethyl benzimidate hydrochloride as the starting material in place of benzamidine salts under optimized conditions, and corresponding products were observed in good yields (55–65%).

**Scheme 30:**
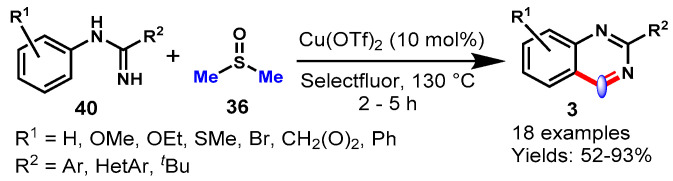
Synthesis of quinazolines from amidines and DMSO.

In 2013, Lv et al. developed an efficient Cu(OTf)_2_-catalyzed one-pot reaction between amidines (**40**) and DMSO (**36**) through the direct oxidative amination of N-H bonds with methyl C(sp^3^)^-^H bonds followed by intramolecular C-C bond formation (Scheme 30) [84]. The generality of the intermolecular annulation reaction was examined by varying different functional groups, including EWGs and EDGs, on the aromatic ring of nitrile moieties, producing the respective quinazolines in good to excellent yields (52–93%). However, cyclic amidine substrates failed to produce the target product under stabilized reaction conditions. Moreover, different carbon sources such as *N*,*N*-dimethylacetamide (DMA), *N*-methyl-2-pyrrolidone (NMP) and tetramethylethane-1,2-diamine (TMEDA) were also found to be effective for the annulation reaction. Notably, *N*,*N*-diethyl-formamide (DEF) and *N*,*N*-diethylacetamide (DEA) did not provide the target product under similar reaction conditions. The kinetic deuterium isotope effect was also analyzed to establish the reaction mechanism.

**Scheme 31:**
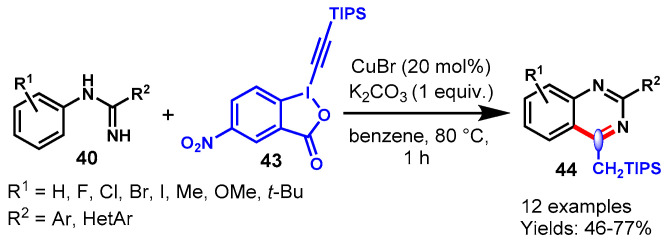
CuBr-catalyzed synthesis of quinazoline from *N*-phenylbenzamidines.

The CuBr-catalyzed synthesis of 2-phenyl-4-[(triiso-propylsilyl)methyl]quinazolines (**44**) via the reaction of *N*-phenylbenzamidines (**40**) with 5-nitro-1-[(triisopropylsilyl)ethynyl]-1,2-benziodoxol-3(1*H*)-one (**43**) in benzene at 80 °C (Scheme 31) has also been presented [85]. This reaction was adequately explored with a broad range of substituted *N*-phenylbenz-amidines containing EWGs and EDGs with 5-nitro-1-[(triisopropylsilyl)-ethynyl]-1,2-benziodoxol-3(1*H*)-one under optimized conditions to afford the corresponding products in moderate to good yields (46–77%). In addition, desilylaion of 2-phenyl-4-(triisopropylsilyl)methyl-quinazoline was carried out in the presence of TBAF in THF-AcOH (20:1) at room temperature, which led to the production of quinazoline in 76% yield via the removal of the TIPS group.

**Scheme 32:**
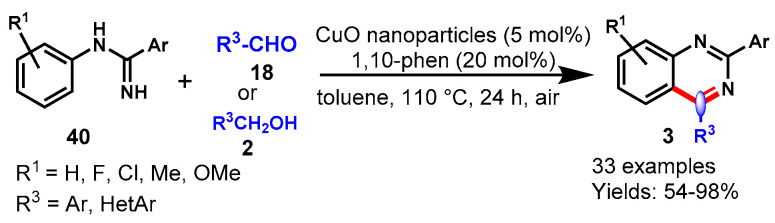
CuO nanoparticles catalyzed synthesis of quinazoline.

In 2014, Wang and team developed a CuO nanoparticle-catalyzed aerobic oxidative coupling reaction of *N*-arylamidines (**40**) and aromatic alcohols (**2**) or aldehydes (**18**) for the synthesis of quinazoline derivatives using 1,10-phen as a ligand in toluene at 110 °C (Scheme 32) [86]. *N*-arylamidines containing both EDGs and EWGs showed good to excellent yields and no significant substitution effect was observed under optimized reaction conditions. A variety of aromatic aldehydes bearing several functional groups such as methyl, methoxy, chloro, nitro and cyano were well tolerated and furnished the respective quinazolines in good yields. Moreover, this methodology worked equally well with heteroaromatic aldehydes under similar reaction conditions. Unfortunately, this reaction did not work effectively with aliphatic aldehydes. Furthermore, the authors extended their work for the synthesis of quinazoline derivatives by using benzyl alcohol as a starting material. There were some advantageous features of this approach that make it attractive for the synthesis of quinazolines, such as it being base- and oxidant-free, as well as the good recyclability of the catalyst without a significant loss of catalytic activity.

**Scheme 33:**
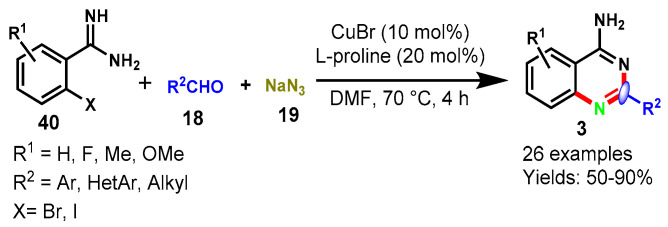
Synthesis of 4-amino quinazoline from halobenzimidamides and aldehydes.

In 2017, a CuBr-catalyzed one-pot tandem strategy was described by Yang et al. for the synthesis of 4-aminoquinazoline (**3**) derivatives using readily available 2-iodobenzimidamide or 2-bromobenzimid-amides (**40**), aldehydes (**18**), and NaN_3_ (**19**) in DMF at 70 °C (Scheme 33) [87]. Different aldehydes, including aliphatic, aromatic, and heteroaromatic, reacted well with various substituted benzimidamides under standard reaction conditions to furnish the corresponding 4-amino quinazoline in 50–90% yields. Mechanistically, the reaction involved a consecutive process, a copper-catalyzed S_N_Ar substitution, reduction, cyclization, and oxidation followed by tautomerization.

**Scheme 34:**
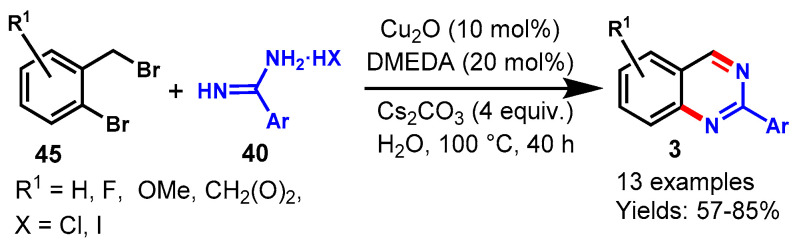
Synthesis of 2-arylquinazoline from ortho-bromobenzylbromides and amidines.

In 2012, Beifuss and team achieved a Cu_2_O-catalyzed one-pot tandem approach for the synthesis of aryl substituted quinazolines from readily available ortho-halobenzylbromides and benzamidines using DMEDA as an additive and Cs_2_CO_3_ as a base in the green solvent at 100 °C (Scheme 34) [88]. Various substituted ortho-halobenzylbromides (**45**) reacted efficiently with benzamidines (**40**) bearing Me and Cl functional groups on the aryl ring and afforded the corresponding substituted quinazolines in good to excellent yields ranging from 57 to 85%.

**Scheme 35:**
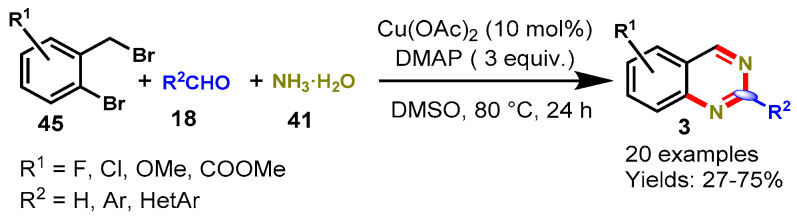
Synthesis of quinazolines from 2-bromobenzyl bromides and aldehydes.

In 2014, Fan et al. described Cu(OAc)_2_-catalyzed one-pot tandem reactions between 2-bromobenzyl bromides (**45**), aldehydes (**18**), and aqueous ammonia (**41**) using DMAP as an additive and DMSO as a solvent at 80 °C (Scheme 35) [89]. Mechanistically, the reaction proceeded through Cu(II)-catalyzed amination, condensation, and intramolecular nucleophilic cyclization, followed by aromatization. A variety of aromatic aldehydes bearing various functional groups, including bromo, chloro, fluoro, nitro, cyano, and trifluoromethyl, were well tolerated by 2-bromobenzyl bromides bearing either electron-donating or electron-withdrawing substituents under optimized reaction conditions. However, the reaction did not work with acetaldehyde and phenylacetaldehyde under similar reaction conditions.

**Scheme 36:**
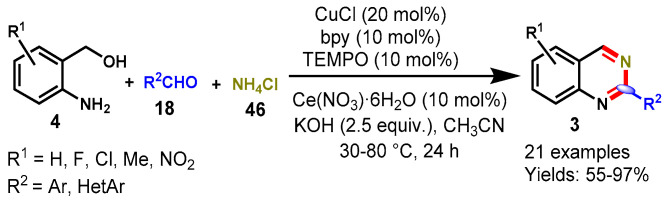
Synthesis of 2-aryl quinazoline from (2-aminophenyl)methanols and aldehydes.

In 2013, Chen et al. described a CuCl-catalyzed tandem reaction between (2-aminophenyl)methanols (**4**), aldehydes (**18**), and ammonium chloride (**46**) in the presence of cerium nitrate hexahydrate, KOH, and TEMPO at 80 °C (Scheme 36) [90]. The developed protocol proceeded smoothly and tolerated a variety of functional groups on the aryl ring of aldehyde, including methoxy, fluoro, chloro, bromo, nitro, formacyl, and trifluoromethyl. In addition, the aldehydes bearing EWGs (F, Cl, Br) produced a higher yield than EDG (Me, OR)-bearing analogs. The authors also scaled up the present synthetic route at the 20 mmol scale and provided 2-phenylquinazoline in an 86% yield. Mechanistically, the reaction involved the oxidation of 2-aminobenzylalcohols to 2-aminobenzaldehydes under a CuCl/2,2′-bipyridine(bpy)/TEMPO catalytic system. Subsequently, the reaction of 2-aminobenzaldehyde with aldehydes and NH_4_Cl provided the cyclized product dihydroquinazoline, which upon aromatization provided the quinazoline derivatives in good to excellent yields (55–97%).

**Scheme 37:**
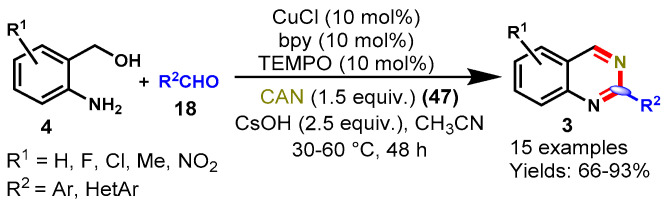
Synthesis of quinazoline from (2-amino-phenyl)methanols and aldehydes.

In 2013, Xia and colleagues demonstrated a CuCl-catalyzed one-pot tandem multi-component approach for the synthesis of quinazolines from (2-aminophenyl)methanols (**4**), aldehydes, and ceric ammonium nitrate (CAN) (**47**) by using CsOH as a base in acetonitrile at 30–60 °C (Scheme 37) [91]. A diverse range of aldehydes including aromatic and hetero-aromatic were well tolerated by different functional groups containing (2-aminophenyl)-methanols, including methyl, fluoro, chloro, and nitro under standard reaction conditions and afforded the corresponding functionalized quinazolines in good to excellent yields (66–93%). Moreover, the authors also explained that the (2-aminophenyl)methanols bearing an electron-donating substituent produced a slightly higher yield than those bearing an electron-withdrawing substituent.

**Scheme 38:**
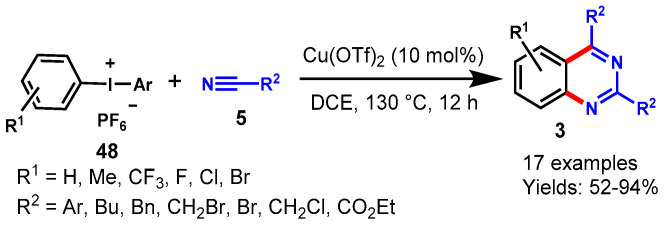
Regio-selective synthesis of quinazolines from diaryliodonium salts and nitriles.

In 2013, Li and colleagues reported a one-pot approach for the regioselective synthesis of substituted quinazolines through [2 + 2 + 2] cascade annulation of diaryliodonium salts (**48**) with nitriles (**5**) using Cu(OTf)_2_ as a catalyst in DMSO at 130 °C (Scheme 38) [45]. A range of aromatic and aliphatic nitriles, including 1-naphthyl and 2-thienyl nitriles, reacted smoothly with diaryliodonium salts and afforded the respective 2,4-diaryl quinazolines in 52–90% yields. However, ethyl cyanoformate (NCCO_2_Et) and diethyl cyanphosphate (NCPO(OEt)_2_) failed to provide the desired product, presumably because of their electron deficiency. Moreover, the authors extended their approach to the synthesis of quinazolines by using two different nitriles through one-pot sequential addition of nitriles, providing the respective quinazoline derivatives in 55–72% yields.

**Scheme 39:**
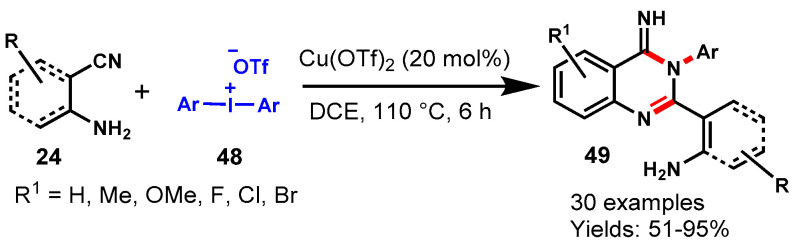
Cu(OTf)_2_-catalyzed synthesis of quinazoline from diaryliodonium salt.

In 2014, Wen and team achieved Cu(OTf)_2_-catalyzed tandem assembly for the synthesis of quinazolin-4(3*H*)-imine (**49**) through the reaction of ortho-cyanoanilines (**24**) and diaryliodonium salts (**48**) in DCE at 110 °C (Scheme 39) [92]. The reaction was satisfactorily explored with a broad range of substituted ortho–cyanoanilines with diaryliodonium salt containing a range of functional groups such as methyl, methoxy, fluoro, chloro, bromo, trifluoromethyl on the aryl ring, and all the substrates reacted smoothly and produced the corresponding quinazolines in moderate to excellent yields (51–95%). In addition, the authors also tested the unsymmetric diaryliodonium salt *p*-CF_3_Ph-I^+^-Mes for the synthesis of quinazoline. Notably, the mesityl group was transferred and the mesityl-containing quinazoline was produced as a major product.

**Scheme 40:**
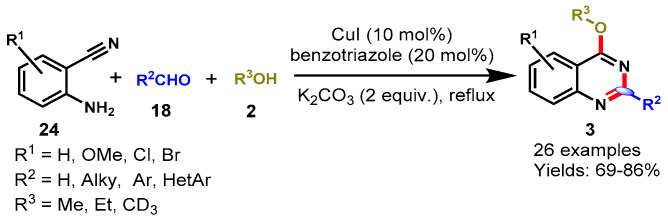
CuI-catalyzed synthesis of *o*-protected-4-hydroxyquinazolines.

In 2014, a unique one-pot strategy to access *o*-methoxy-protected quinazolines was introduced by Ahmed’s group via the copper-benzotriazole (Cu-BtH)-catalyzed intramolecular electrophilic cyclization of *N*-arylimines through the reaction of 2-aminobenzonitriles (**24**) and aldehydes (**18**) in the presence of methanol (**2**) (Scheme 40) [93]. The reaction was adequately explored with a broad range of substituted 2-aminobenzonitriles and aldehydes, containing EWGs and ERGs, and all the substrate reacted smoothly and produced the respective products in good to excellent yields (41–88%). However, aryl and heteroaryl aldehydes provided improved reaction yields as compared to aliphatic aldehydes. Further, the authors extended the approach and synthesized *o*-ethoxy-protected quinazolines by using ethanol as a solvent under dry conditions in the presence of molecular sieves.

**Scheme 41:**
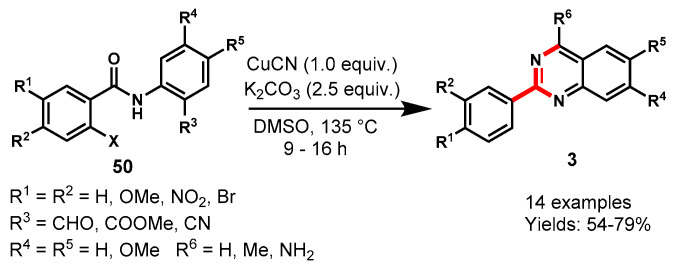
Synthesis of quinazolines from ortho-substituted 2-halo-*N*-arylbenzamides.

In 2015, Yao’s group demonstrated a CuCN-catalyzed one-pot approach for the synthesis of 2-aryl quinazolines and tetracyclic isoindolo[1,2-*a*]-quinazoline using K_2_CO_3_ as a base in DMSO at 135 °C (Scheme 41) [94]. Mechanistically, the reaction proceeded through CuCN-catalyzed cyanation followed by the rearrangement of ortho-substituted 2-halo-*N*-arylbenzamides (**50**). Moreover, changing the reaction solvent such as 1,4-dioxane led to the formation of tetracyclic isoindolo[1,2-*a*]quinazoline as a major product. Further, base-catalyzed cleavage of tetracyclic isoindolo[1,2-*a*]quinazolines derivatives produced the respective 2-arylquinazolines as a major product. A variety of quinazolines derivatives like 2-phenylquinazolin-4-amine, 4-methyl-2-phenylquin-azoline, and long-chain 2-phenyl-4-styrylquinazoline were observed under optimized reaction conditions with 54–79% yields.

**Scheme 42:**
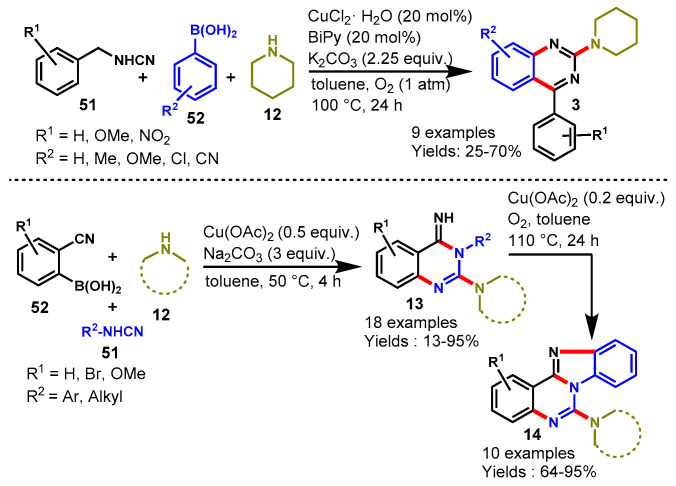
Synthesis of quinazolines from cyanamides and boronic acid.

In 2015, Neuville’s group developed CuCl_2_-promoted one-pot three-component rapid assembly for the synthesis of 2-aminoquinazolines from the reaction of easily available cyanamides (**51**), arylboronic acids (**52**), and amines (**12**) using K_2_CO_3_ as a base and 2,2′-bipyridine as ligand under O_2_ atmosphere (1 atm) at 100 °C (Scheme 42) [95]. In this approach, copper promotes the formation of three bonds, including two C-N bonds and one bond through a C-H functionalization event. In 2019, the same group explored their work for the synthesis of functionalized quinazolin-4(*H*)-imines (**14**) from cyanamides (**51**), 2-cyanoarylboronic acids (**52**), and amines (**12**) using Cu(OAc)_2_ as a catalyst and Na_2_CO_3_ as a base in toluene at 50 °C [96]. Different functional groups containing 2-cyanoboronic acid and cynamides reacted efficiently and provided the functionalized quinazolin-4(*H*)-imines in 13–95% yields. Moreover, the authors explored the utility of the reaction and synthesized benzimidazo[1,2-*c*]quinazolines through a copper-catalyzed C-H amination process.

**Scheme 43:**
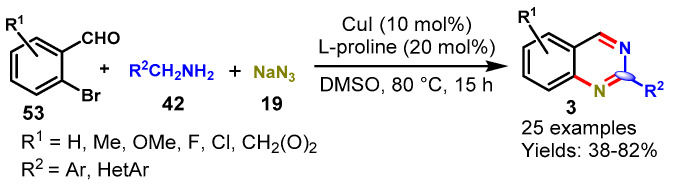
Synthesis of quinazolines from 2-bromobenzaldehydes and benzylamines.

In 2016, Wu and the team described a CuI-catalyzed tandem multi-component strategy for the synthesis of quinazoline derivatives from 2-bromobenzaldehyde (**53**), benzylamine (**42**), and sodium azide (**19**) in DMSO at 80 °C. Mechanistically, the reaction involved sequential copper-catalyzed S_N_Ar, oxidation/cyclization, and a denitrogenation process (Scheme 43) [97]. A series of aldehydes including aromatic and heteroaromatic reacted smoothly with benzylamines bearing EDGs (Me, OMe, OH) and EWGs (F, Cl, Br) under optimized conditions to provide functionalized quinazoline in 38–82% yields. Notably, naphthalen-1-ylmethanamine, pyridin-3-ylmethanamine also worked well under optimized conditions and furnished the corresponding products in 42% and 75% yields, respectively. Additionally, three C-N bonds were constructed in a one pot tandem fashion under mild reaction conditions.

**Scheme 44:**
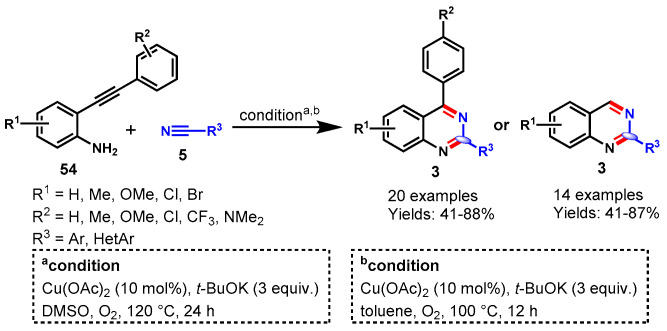
Synthesis of quinazolines from 2-(phenyl-ethynyl)aniline and benzonitriles.

In 2018, Jiang and team developed a Cu(OAc)_2_-catalyzed one-pot reaction of 2-(phenylethynyl)aniline (**54**) and benzonitriles (**5**) for the synthesis of quinazolines by using molecular oxygen as a sole oxidant and *t*-BuOK as a base at 120 °C in DMSO (Scheme 44) [98]. Interestingly, the author observed different products when the solvent switched from DMSO to toluene. The reaction showcased a wide range of substituent tolerance with various benzonitriles and 2-ethynylanilines, providing a gallery of quinazolines in moderate to excellent yields (41–88%). The reaction proceeded through the effective cleavage of the C-C triple bond and C-C and C-N bond formation in a one-pot operation. In addition, the synthesized compounds displayed aggregation-induced emission effects, good fluorescence quantum yield, and lifetime decay, which enhanced the value of quinazoline analogs in material science for future aspects.

**Scheme 45:**
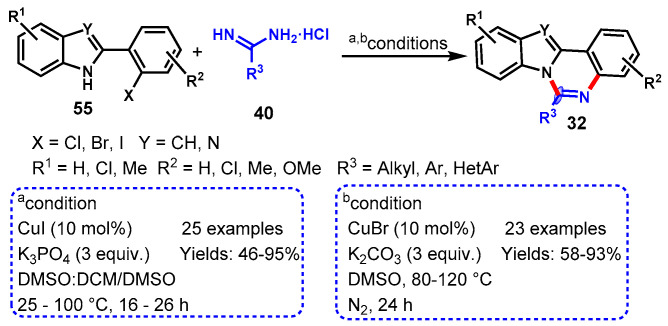
(**a**) Synthesis of quinazolines from 2-(2-halo-phenyl)benzimidazoles/indoles and amidines; (**b**) Cu-catalyzed regioselective approach for the synthesis of 1*H*-indolo[1,2-*c*]quinazolines.

In 2011, a one-pot cascade method to access fused quinazolines (**32**) was described by Fu’s group using 2-(2-halophenyl)-*1H*-benzo[*d*]imidazole (**55**) with amidines or guanidines (**40**) through CuI-catalyzed Ullmann-type C-N coupling followed by intramolecular nucleophilic attack of NH on amidines or guanidine carbon. The various functional groups decorating benzimidazoles and amidine or guanidine substrates worked effectively under the stabilized conditions to furnish the corresponding fused quinazolines in moderate to excellent yields (46–95%) (Scheme 45a) [99]. Moreover, the authors also described the ortho-substituent effect of halogen on benzimidazole moieties. Substituted 2-(2-bromophenyl)benzoimidazoles reacted well at room temperature, while in the case of 2-(2-chlorophenyl)benzoimidazole, the temperature was increased up to 100 °C due to the lower reactivity of aryl chloride for C-N coupling reactions.

As a continuation of their research, in 2012, Fu’s group demonstrated another Cu-catalyzed regioselective approach for the synthesis of 1*H*-indolo[1,2-*c*]quinazolines from the reaction of 2-(2-halophenyl)indoles and amidines using K_2_CO_3_ as a base in DMSO under a nitrogen atmosphere (Scheme 45b) [100]. Various 2-(2-halophenyl)indoles reacted smoothly with electronically variable amidines under optimized conditions, yielding the respective products in good to excellent yields (58–93%). However, aliphatic amidines showed slightly higher yields than aromatic ones. Interestingly, more highly regioselective products were observed through N-1 cyclization over C-3 cyclization of indoles.

**Scheme 46:**
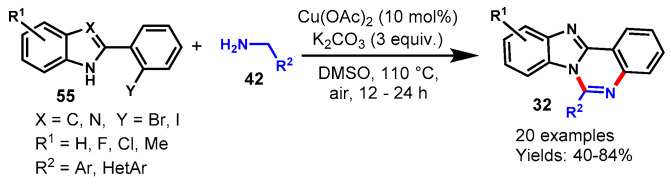
Cu-catalyzed synthesis of quinazolines from2-(2-bromophenyl)-*1H*-benzo[*d*]imidazole.

In 2012, Sang et al. reported a Cu(OAc)_2_-catalyzed sequential Ullmann-type *N*-arylation and aerobic oxidative C-H amination between 2-(2-halophenyl)-*1H*-benzo[*d*]imidazole/indoles (**55**) and arylmethanamines (**42**) for the synthesis of fused quinazolines using K_2_CO_3_ as a base in DMSO at 110 °C (Scheme 46) [101]. The scope of this methodology was examined under optimized conditions, and aromatic/heteroaromatic methanamines reacted smoothly with 2-(2-halo-phenyl)-*1H*-benzimidazoles/indoles and produced the corresponding quinazoline derivatives (**32**) in 40–84% yields. Naphthyl-substituted methanamines were also tolerated well under stabilized reaction conditions. In particular, aryl iodides showed higher reactivity than arylbromide for Ullmann-type *N*-arylation.

**Scheme 47:**
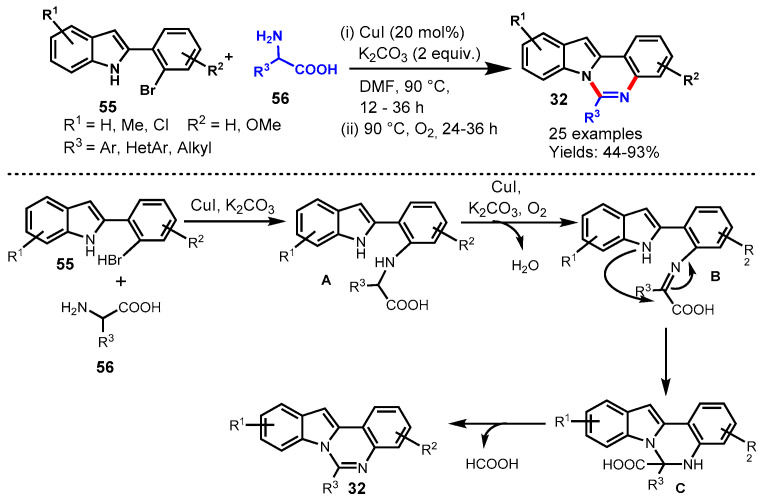
Synthesis of indolo[1,2-*c*]quinazolines from 2-(2-bromophenyl)-1*H*-indole and α-amino acid.

In 2013, Fu et al. demonstrated a CuI-catalyzed aerobic oxidative approach for the synthesis of indolo[1,2-*c*]quinazolines from the reaction of 2-(2-bromophenyl)-1*H*-indole (**55**) with α-amino acid (**56**) using K_2_CO_3_ as base in DMF under oxygen atmosphere (Scheme 47) [102]. Different α-amino acids such as aliphatic, aromatic, and heteroaromatic were compatible with 2-(2-bromophenyl)-1*H*-indole under standard reaction conditions and delivered the diversely substituted indolo[1,2-*c*]quinazolines with moderate to excellent yields (44–93%). Notably, the developed protocol also worked well with pipecolinic acid under stabilized conditions and afforded the corresponding product in 64% yields. In addition, the authors also successfully applied a developed protocol for the synthesis of benzo[4,5]imidazo[1,2-*c*]quinazolines and pyrazolo[1,5-*c*]quinazolines under similar reaction conditions. Mechanistically, the reactions involved Cu-catalyzed *N*-arylation and aerobic oxidative dehydrogenation followed by intramolecular cyclization.

**Scheme 48:**
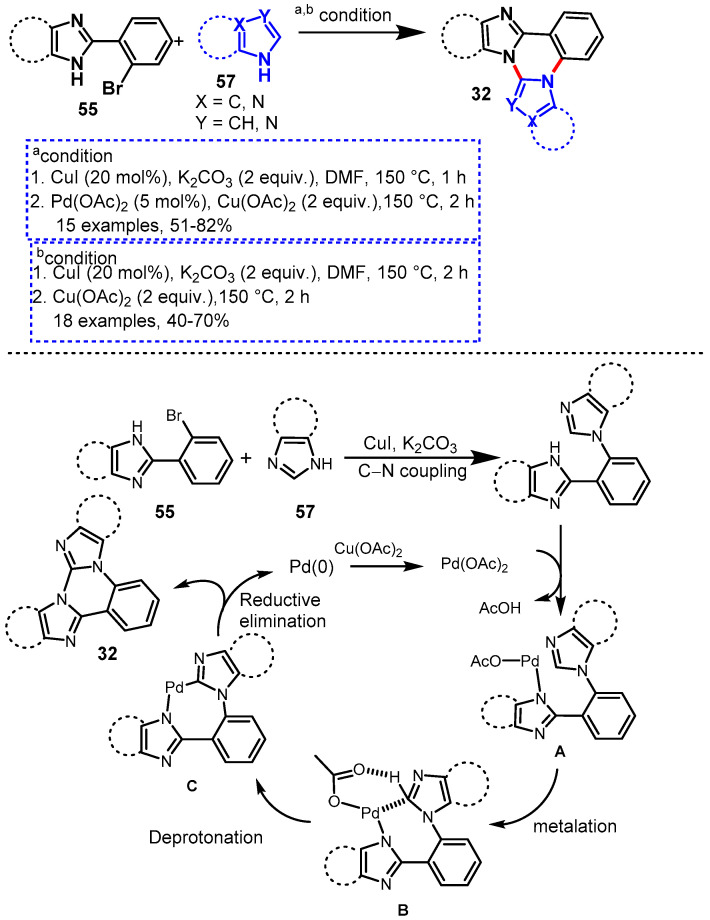
(**a**) Cu/Pdcatalyzed synthesis of imidazo/benzimidazo[1,2-*c*]quinazolines; (**b**) Coppercatalyzed one-pot sequential approach for the synthesis of azole-fused quinazolines.

In 2014, Nandwana et al. described a Pd/Cu co-catalyzed one-pot sequential approach for the synthesis of novel azole-fused quinazolines (**32**) from the reaction of 2-(2-bromophenyl)-1*H*-imidazole/-benzimidazoles (**55**) and different azole derivatives (**57**) such as 1*H*-imidazole, 1*H*-benzimidazole, and 1*H*-1,2,4-triazole. The mechanism of this reaction involved a copper-catalyzed Ullmann type C-N coupling reaction followed by a Pd(OAc)_2_/Cu(OAc)_2_ catalyzed cross-dehydrogenative coupling reaction (Scheme 48a) [103]. The developed protocol worked well under optimized conditions and delivered the corresponding *N*-fused tetra-, penta- and hexa-cyclic frameworks in good to excellent yields (52–81%).

Further, in 2019, the same authors improved the reaction conditions and targeted fused quinazolines were achieved under copper-catalyzed conditions. The synthesized compounds were tested for their in vitro antibacterial activity against three Gram-negative (*Escherichia coli*, *Pseudomonas putida*, *Salmonella typhi*) and two Gram-positive (*Bacillus subtilis*, *Staphylococcus aureus*) bacteria. Among all tested compounds, three compounds exhibited promising MIC values (4–8 µg/mL). The synthesized compounds were also evaluated for their in vitro antifungal activity and showed pronounced antifungal activity (MIC values 8–16 µg/mL) against both strains (Scheme 48b) [104]. Furthermore, the synthesized compounds were also evaluated for their hemolytic activity, showing a negligible toxicity profile toward human blood cells.

**Scheme 49:**
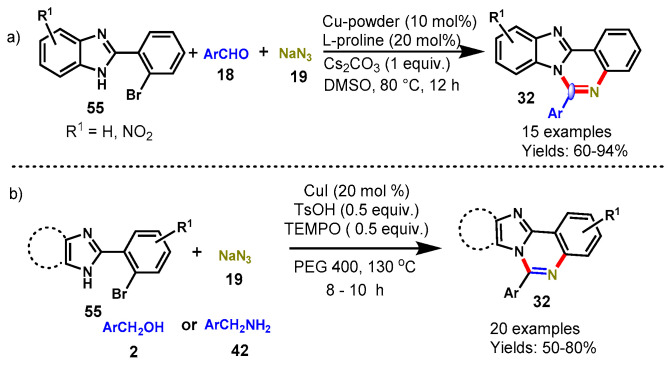
(**a**) Synthesis of benzimidazo[1,2-*c*]quinazolines from (2-halophenyl)benzimidazoles and aldehydes; (**b**) One-pot three-component strategy for the synthesis of imidazo[1,2-*c*]quinazolines and benzimidozo[1,2-*c*]quinazolines.

In 2015, a copper-catalyzed one-pot multi-component reaction demonstrated by Kumar’s group by using 2-(2-halophenyl)benzimidazoles (**55**), aldehydes (**18**), and sodium azide (**19**) with L-proline as a ligand and CS_2_CO_3_ as a base in DMSO at 80 °C (Scheme 49a) [105]. This reaction involved three consecutive C-N bond formations: the azidation of arylhalide with sodium azide, then the in situ conversion of aryl azide to arylamine through reduction, followed by condensation and oxidative cyclization, providing the respective benzimidazo[1,2-*c*]quinazoline in good to excellent yields (60–94%). The generality of the reactions was examined by varying different aromatic aldehydes, and EDGs on the phenyl ring provided better yields than ERGs. Interestingly, 1-naphthaldehyde also worked smoothly under optimized conditions and produced the corresponding product with a 94% yield. Moreover, a radical trapping experiment was conducted by using 2,2,6,6-tetramethylpiperidine-1-oxyl (TEMPO), which revealed the radical mechanism for the proposed reactions.

Recently, Nandwana et al. described a one-pot three-component strategy for the synthesis of imidazo[1,2-*c*]quinazolines and benzimidozo[1,2-*c*]quinazolines from 2-(2-bromophenyl)-1*H*-imid-azoles/benzimidazoles from benzyl alcohol or benzylamine as a benzaldehyde surrogate and sodium azide as a nitrogen source by using green solvent (PEG 400) (Scheme 49b) [106].

**Scheme 50:**
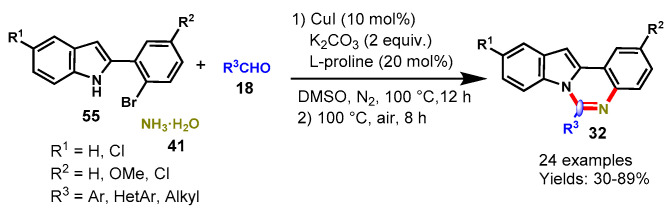
Synthesis of quinazolines from 2-(2-bromo-aryl)-1*H* indoles and aldehydes.

In 2015, Fan and colleagues described copper-catalyzed one-pot cascade reactions of 2-(2-bromo-aryl)-1*H* indoles (**55**), aldehydes (**18**), and aqueous ammonia (**41**) for the synthesis of indolo[1,2-*c*]quinazoline using K_2_CO_3_ as a base and L-proline as a ligand in DMSO under a nitrogen atmosphere (Scheme 50) [107]. Diversely substituted aldehydes with ERGs (Me, OMe) and EWGs (CF_3_, F, Cl, Br) reacted well with 2-(2-bromoaryl)-1*H* indoles under optimized conditions to provide the desired products in moderate to good yields. Notably, 1-naphthaldehyde and thiophene-2-carbaldehyde, cinnamaldehyde, and butyraldehyde were also well tolerated under stabilized reaction conditions. Interestingly, when the reaction was performed under acidic conditions, 11*H*-indolo[3,2-*c*]quinolines was observed as a product through C-C coupling reactions.

**Scheme 51:**
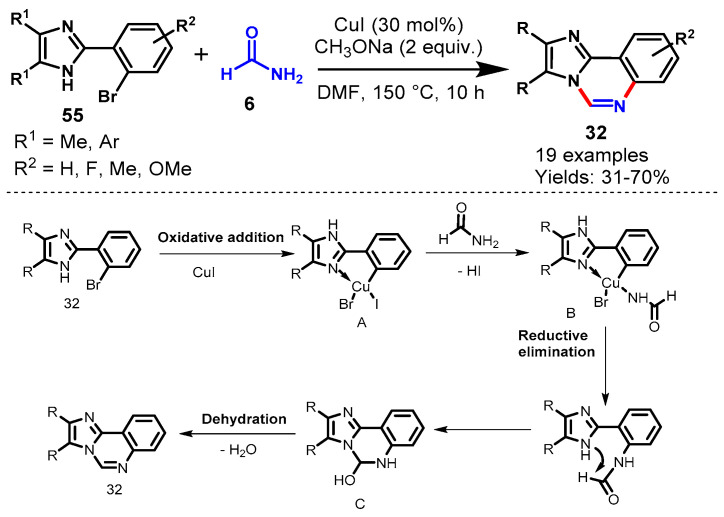
CuI-catalyzed tandem Ullmann type C-N coupling and dehydrative cyclization.

In 2016, we developed a CuI-catalyzed one-pot tandem approach between 2-(2-bromophenyl)-1*H*-imidazoles (**55**) and formamide (**6**) for the synthesis of imidazo[1,2-*c*]quinazolines (Scheme 51) [108]. Mechanistically, the reaction proceeded through copper-catalyzed Ullmann-type C-N coupling and intramolecular nucleophilic addition followed by dehydrative cyclization. The generality of the tandem reaction was investigated by varying different functional groups like Me, OMe, and F on the aryl ring of 2-(2-bromophenyl)-1*H*-imidazoles, which reacted efficiently with formamide under optimized conditions to produce respective quinazolines in 31–70% yields. The developed protocol also worked well at the gram scale with a 71% yield. Moreover, authors also accomplished the tandem reaction with acetamide and benzamide under optimized conditions. Unfortunately, under these reaction conditions, only C-N coupled products were observed; this might be due to the low electrophilicity of the amidecarbonyl group in acetamide and benzamide as compared to formamide. Interestingly, targeted products were observed in 57% and 43%, respectively, under BF_3_.OEt_2_ in DMF at 150 °C.

**Scheme 52:**
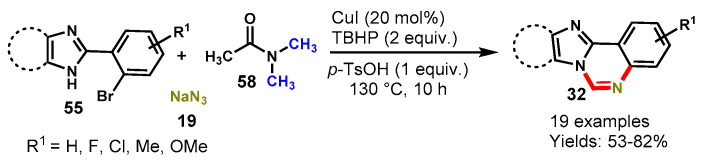
Synthesis of quinazolines through reductive amination and oxidative amination of C(sp^3^)-H bond.

In 2017, Kumar and the team reported an efficient one-pot tandem approach for the synthesis of quinazolines from the reaction of 2-phenyl imidazole/benzimidazole (**55**), NaN_3_ (**19**) as a nitrogen source and DMA (**58**) as a carbon source using TBHP as the oxidant and *P*-TsOH as an additive at 130 °C (Scheme 52) [109].The developed protocol reacted well with 4,5-diaryl-2-(2-bromoaryl)-1*H*-imidazoles/-2-(2-bromophenyl)-*1H*-benzo[*d*]imidazoles with different substituents such fluoro, chloro, methyl and methoxy on aryl rings of imidazole/benzimidazole to produce respective quinazolines in moderate to good yields (53–82%). The radical scavenger experiment was also performed by using TEMPO, suggesting that the reaction mechanism does not involve a free radical pathway. Mechanistically, the reaction involved sequential azidation through S_N_Ar, and reduction, followed by oxidative amination of C(sp^3^)-H bonds of *N,N*-dimethylacetamide. The present method was scaled up to a gram scale, yielding the 2,3-diphenylimidazo[1,2-*c*]quinazoline in 80% yield without a significant loss in reaction efficacy. Furthermore, the authors applied this methodology to the synthesis of quinazolinone and quinoline derivatives. Interestingly, all of the derivatives worked well under similar reaction conditions and afforded the respective products in 37–71% yields.

**Scheme 53:**
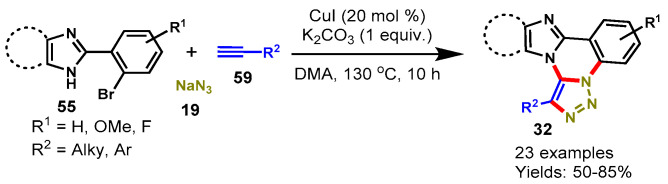
Synthesis of imidazo[1,2-*c*][1,2,3]triazolo[1,5-*a*]quinazolines from 2-(2-bromoaryl)imidazoles.

In 2017, Nandwana et al. described a CuI-catalyzed tandem reaction of 2-(2-bromoaryl)imidazoles/2-(2-bromoaryl)benzimidazoles (**55**), alkynes (**59**), and sodium azide (**19**) for the synthesis of imidazo[1,2-*c*][1,2,3]triazolo[1,5-*a*]quinazolines in DMA (Scheme 53) [110]. Various substituted 2-phenylimidazole/benzimidazole derivatives reacted efficiently with aliphatic and aromatic alkynes under stabilized reaction conditions to provide corresponding fused quinazolines in moderate to good yields (50–85%). However, imidazole bearing NO_2_ functional groups failed to provide the target product under the optimized reaction conditions. Mechanistically, the tandem approach involved copper-catalyzed azide-alkyne cycloaddition (CuAAC) and intramolecular cross dehydrogenative C-N bonding followed by the Ullmann type C-N coupling reaction. In addition, 1-phenyl-indolo[1,2-*c*][1,2,3]triazolo[1,5-*a*]quinazoline was also synthesized from the reaction of 2-(2-bromophenyl)-1*H*-indole with phenylacetylene and NaN_3_ under similar reaction conditions.

**Scheme 54:**
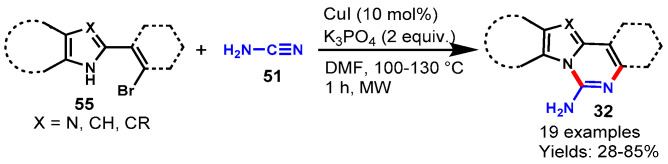
Synthesis of quinazolines from cyanamides under microwave conditions.

In 2017, Cho and team demonstrated a simple and efficient CuI-catalyzed method for the synthesis of benzo[4,5]imidazo[1,2-*c*]quinazolin-6-amines/benzo-[4,5]imidazo[1,2-c]pyrimidin-1-amines (**32**) under microwave irradiation through the reaction of 2-(2-bromophenyl)benzimidazoles/2-(2-bromovinyl)benz-imidazoles (**55**) with cyanamide (**51**) in the presence of K_3_PO_4_ as a base in DMF at 100–130 °C for 1 h (Scheme 54) [111]. Diversely substituted imidazole and benzimidazole reactged well with cyanamide under optimized conditions to provide the respective products in 28–85% yields. Furthermore, the developed protocol extended to the reaction of 2-(2-bromophenyl)indoles and cyanamide under optimized conditions to produce the corresponding indolo[1,2-*c*]quinazolin-6-amines in good yields (50–73%). Mechanistically, the reaction proceeded through copper-catalyzed intermolecular Ullmann type C-N coupling and C-N formative cyclization followed by tautomerization.

**Scheme 55:**
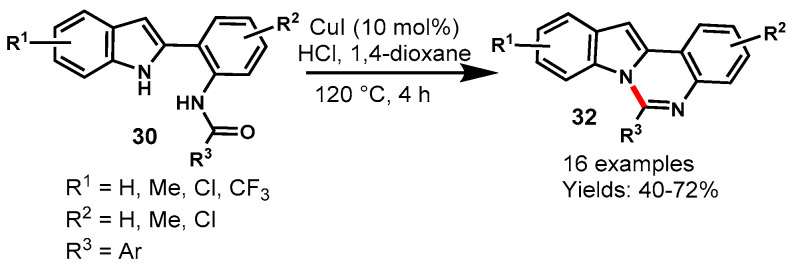
Synthesis of indolo[1,2-*c*]quinazolines from 2-(2-amidoaryl)-1*H*-indoles.

In 2018, Guo et al. described a CuI-catalyzed intramolecular cyclization reaction of 2-(2-amidoaryl)-1*H*-indoles (**30**) for the synthesis of indolo[1,2-*c*]quinazolines (**32**) derivatives in 1,4-dioxane at 120 °C (Scheme 55) [112]. Various indole substrates with an aromatic and aliphatic substituent on the amide unit and EDGs and EWGs attached to the 2-phenyl ring of indoles reacted well under optimized reaction conditions to produce the respective products in 40–72% yields. The mechanism of the reaction involved the nucleophilic addition of indole nitrogen to amidic carbonyl followed by dehydration. In addition, by tuning the reaction parameters such as solvents from 1,4-dioxane to DMF, the authors observed 3*H*-indol-3-one as a major product.

**Scheme 56:**
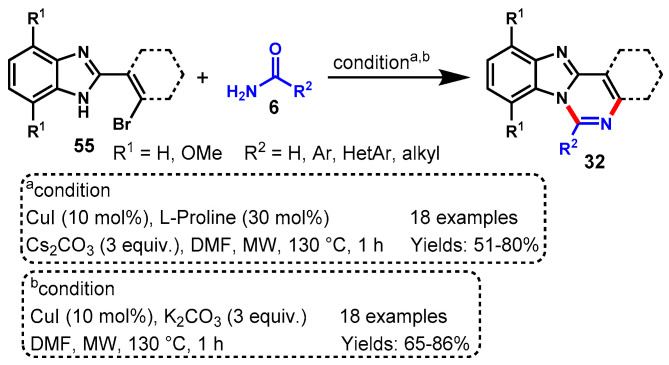
Synthesis of quinazolines from 2-(2-bromoaryl)-benzimidazoles and amides.

In 2018, Chon and team achieved another CuI-catalyzed one-pot assembly for the synthesis of benzo[4,5]imidazo[1,2-*c*]quinazolines under microwave irradiation or usual heating from 2-(2-bromoaryl)benzimidazoles/(2-bromovinyl)-benzimid-azoles and primary amides (**6**) (Scheme 56) [113]. Variously substituted amides, including aliphatic, aromatic, and heteroaromatic, participated well with 2-(2-bromoaryl)-benzimidazoles/(2-bromo-vinyl)-benzimidazoles under stabilized reaction conditions to provide the desired products in moderate to good yields. In addition, when benzo[4,5]-imidazo[1,2-*c*]quinazolines with a methoxy group on the benzimidazole moiety were treated with aqueous ceric ammonium nitrate (CAN) in aqueous acetonitrile at room temperature, all of the derivatives converted into quinazoline- and pyrimidine-fused benzimidazolequinones with 70–89% yields.

**Scheme 57:**
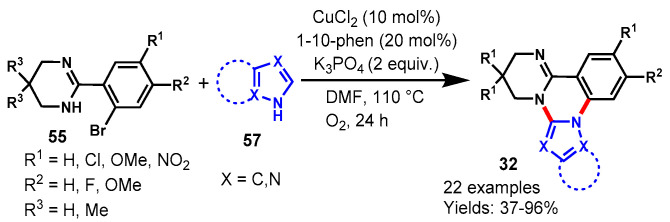
CuCl_2_-catalyzed double C-N coupling reaction for the synthesis of azole-fused quinazolines.

In 2018, Chen et al. described a CuCl_2_-catalyzed double C-N coupling reaction for the synthesis of azole-fused pyrimido[1,2-*c*]quinazolines and imidazo-[1,2-*c*]quinazolines (**32**) in the presence of 1,10-phen as a ligand and K_3_PO_4_ as a base in DMF at 110 °C under an oxygen atmosphere. The developed protocol proceeded through copper-catalyzed Ullmann-type C-N coupling followed by C-H functionalization (Scheme 57) [114]. The scope of the reaction was examined by varying different azoles (**57**) such as benzimidazoles, pyrazoles, and 1,2,4-triazoles which were compatible under optimized conditions and afforded the target products in moderate to excellent yields (37–96%).

**Scheme 58:**
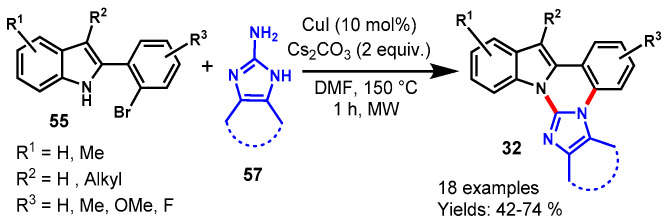
Synthesis of indole fused quinazolines from 2-(2-bromoaryl)indoles and 2-aminoazoles.

In 2020, Cho and team explored CuI-catalyzed trinuclear *N*-fused hybrid scaffolds by using 2-(2-bromoaryl)indoles (**55**) and 2-aminoazoles (**57**) as the starting material under microwave irradiation in DMF at 150 °C (Scheme 58) [115]. 2-(2-Bromoaryl)indole derivatives bearing straight, branched alkyl chains and different functional groups such as Me, OMe, and F on the indole moiety worked well with 2-aminoazoles under stabilized reaction conditions to provide the respective fused quinazolines (**32**) in 42–74% yields. Notably, 2-(2-bromophenyl)indoles bearing the chloro functional group failed to produce target products under optimized conditions and provided the dechlorinated trinuclear *N*-fused hybrid scaffold. Mechanistically, the reaction involved copper-catalyzed C(sp^2^)-N coupling followed by intramolecular cyclo condensation.

**Scheme 59:**
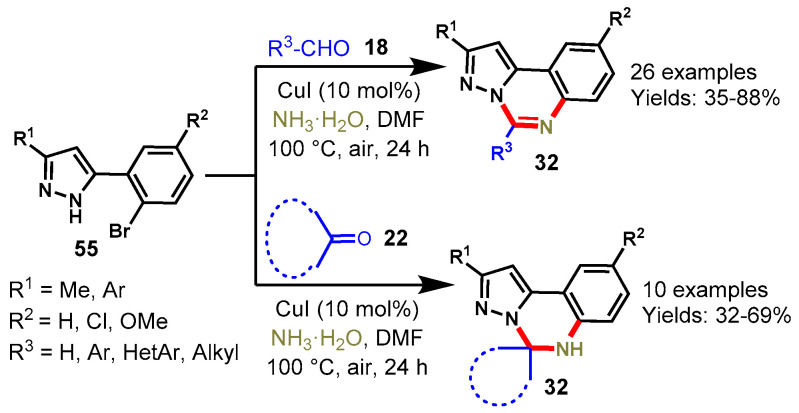
Synthesis of pyrazolo[1,5-*c*]quinazolines from 5-(2-bromoaryl)-1*H*-pyrazoles with aldehydes.

In 2013, Gou and his colleagues developed an efficient method for the synthesis of pyrazolo[1,5-*c*]quinazolines and 5,6-dihydropyrazolo[1,5-*c*]quinazolines via a copper-catalyzed one-pot tandem reaction of 5-(2-bromoaryl)-1*H*-pyrazoles with aldehydes (**18**) and ketones (**22**) in aqueous ammonia under aerobic condition (Scheme 59) [116]. A diverse range of aldehydes including aryl, alkyl, alkenyl, and hetero-aryl reacted smoothly and afforded the corresponding functionalized quinazolines in moderate to good yields (35–88%). Moreover, ketone derivatives also reacted well and provided the respective product in 32–69% yields. In addition, the authors also explored the reaction in a four-component manner by using 1-(2-bromophenyl)-1,3-diones, hydrazine hydrate, carbonyl compounds, and aqueous ammonia under copper-catalyzed conditions and observed the corresponding products in 36–74% yields.

**Scheme 60:**
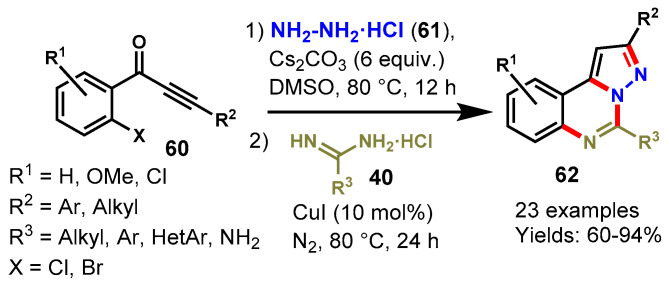
Cu-catalyzed synthesis of pyrazolo[1,5-*c*]-quinazolines.

In 2012, Fu and co-workers described a CuI-catalyzed one-pot sequential approach for the construction of pyrazolo[1,5-*c*]quinazolines (**62**) from readily available 1-(2-halophenyl)-3-alkylprop-2-yn-1-ones (**60**), hydrazine hydrochlorides (**61**), and amidine hydrochlorides (**40**) in DMSO under a nitrogen atmosphere (Scheme 60) [117]. The scope of this methodology was explored by varying different substituents on 1-(2-halophenyl)-3-alkylprop-2-yn-1-ones, which reacted smoothly with aliphatic/aromatic/heteroaromatic amidines under standard reaction conditions and produced the respective products in 60–94% yields. Mechanistically, the reaction involved base-mediated pyrazole formation then copper-catalyzed *N*-arylation followed by intramolecular nucleophilic reactions.

**Scheme 61:**
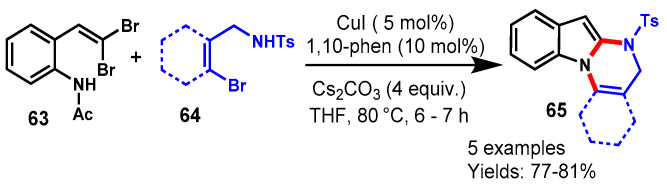
Synthesis of indolo[1,2-*a*]quinazolines from gem-dibromovinylanilides and *N*-tosyl-o-bromobenz-amides.

In 2013, Kiruthika et al. demonstrated a CuI-catalyzed one-pot protocol for the rapid synthesis of tetrahydroindolo[1,2-*a*]quinazolines through the reaction of gem-dibromovinylanilides (**63**) with *N*-tosyl-*o*-bromobenzamides (**64**) using 1,10-phen as a ligand and Cs_2_CO_3_ as a base in THF at 80 °C (Scheme 61) [118]. Different *N*-tosyl-*o*-bromobenzamides reacted smoothly with gem-dibromovinylanilides under optimized conditions and delivered the respective products with 77–81% yields. Moreover, the tosyl group was removed under basic conditions and the corresponding indolo[1,2-*a*]quinazolines were observed in 87–92% of yields.

**Scheme 62:**
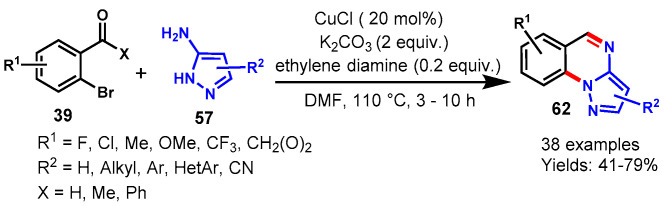
Synthesis of quinazolines from 2-bromo-benzaldehydes/ketones and 5-aminopyrazoles.

In 2014, a CuCl-catalyzed one-pot tandem approach has described by Fan group for the synthesis of pyrazolo[1,5-*a*]quinazolines (**62**) from 2-bromo-benzaldehydes/ketones (**39**) and 5-aminopyrazoles (**57**) using ethylenediamine as ligand and K_2_CO_3_ a base in DMF at 110 °C. (Scheme 62) [119]. Various functional groups bearing 2-bromobenzaldehydes such as methyl, methoxy, trifluoromethyl, and halides on aryl ring were well tolerated with diversely substituted (Me, CN, Ph, cyclopropane, thiophene) 5-aminopyrazoles under optimized reaction conditions and produced the corresponding fused quinazolines in 41–83% yields. Notably, 2-bromonicotinaldehyde was also found a suitable substrate for this transformation, providing pyrazolo[1,5-*a*]pyrido[3,2-*e*]pyrimidine in 79% yield. In addition, 2-bromophenyl methyl ketones and 2-bromophenyl phenyl ketones were also participated well with 5-aminopyrazoles under the same reaction conditions to afford corresponding products in 43–69% yields. Mechanistically, the reaction proceeded through imine formation followed by a copper-catalyzed intramolecular C-N coupling reaction.

**Scheme 63:**
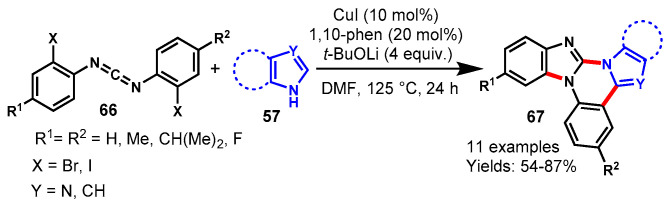
CuI-catalyzed tandem approach for the synthesis of azole–fused quinazolines.

In 2014, Lv and colleagues reported an efficient approach for the assembly of azole-fused quinazolines (**67**) through the Cu-catalyzed domino addition/double cyclization process using 1,10-phen as a ligand and *t*-BuOLi as a base in DMF (Scheme 63) [120]. Mechanistically, the reaction involved nucleophilic addition, intramolecular C-N coupling, and intramolecular sp^2^ C-H arylation of azole with bis-(*o*-haloaryl)-carbodiimide (**66**). The bis-(*o*-iodo-phenyl)carbodiimides bearing electron-donating groups (*p*-Me) and electron-withdrawing groups (*p*-F) on the aryl rings both reacted smoothly with a variety of azoles (**57**) such as imidazole, benzimidazoles, pyrazole, and indoles under stabilized reaction conditions, delivering the corresponding desired products in 54–87% yields. However, benzimidazole derivatives showed higher reactivity than other azoles. In addition, indole derivatives also reacted well under similar reaction conditions to produce the corresponding benzo[4,5]imidazo[1,2-*a*]indolo-[1,2-*c*]-quinazolines in 62–70% yields via addition and coupling followed by the direct C2-arylation process.

**Scheme 64:**
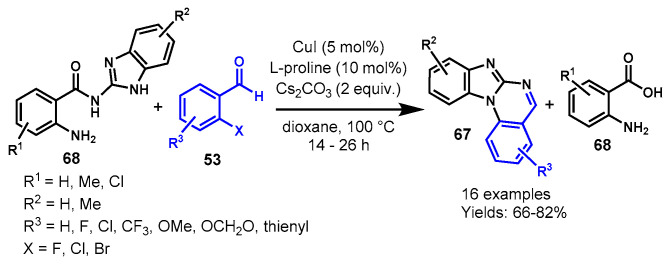
Synthesis of quinazolines from *N*-(2-benzimidazolyl)-2-aminobenzamide and 2-halo benzaldehyde.

In 2014, Wang group demonstrated a CuI-catalyzed tandem approach for the generation of benzimidazo[1,2-*a*]quinazolines (**67**) from the reaction of *N*-(2-benzimidazolyl)-2-aminobenzamide (**68**) with 2-halobenzaldehyde (**53**) using L-proline as a ligand and Cs_2_CO_3_ as a base in 1,4-dioxane at 100 °C (Scheme 64) [121]. *N*-(2-Benzimidazolyl)-2-amino-benzamides and 2-halobenzaldehydes decorated with EWGs and ERGs worked efficiently under optimized conditions to afford the corresponding products in 66–82% yields along with substituted 2-aminobenzoic acid. Moreover, the reaction also reacted smoothly with 3-bromothiophene-2-carbaldehyde and produced the benzo[4,5]-imidazo[1,2-*a*]thieno[2,3-*e*]pyrimidine in 82% yield. Mechanistically, the reaction proceeded through sequential copper-catalyzed Ullmann *N*-arylation followed by two C-N bond cleavage reactions.

**Scheme 65:**
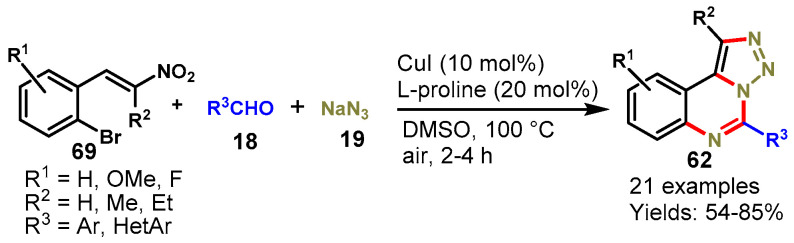
Synthesis of quinazolines from (*E*)-1-bromo-2-(2-nitrovinyl)benzenes and aldehydes.

In 2015, Jia et al. developed a CuI-catalyzed tandem multi-component approach for the synthesis of triazolo[1,5-*c*]quinazolines (**62**) from the reaction of (*E*)-1-bromo-2-(2-nitrovinyl)benzenes (**69**), aldehydes (**18**), and sodium azide (**19**) in the presence of L-proline in DMSO at 100 °C. Various functional group-containing aromatic aldehydes like ERGs and EWGs demonstrated good compatibility with (*E*)-1-bromo-2-(2-nitrovinyl)benzenes under standard reaction conditions and produced the target products with moderate to excellent yields (54–85%). The tandem approach involved consecutive [3 + 2] cycloaddition, copper-catalyzed S_N_Ar, reduction, cyclization, and oxidation sequences. Notably, sodium azide acted as a dual nitrogen source for the construction of fused quinazolines (Scheme 65) [122].

**Scheme 66:**
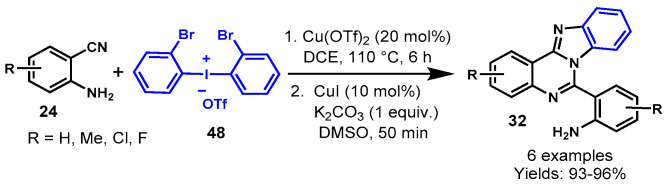
Synthesis of quinazolines from o-cyanoanilines and diaryliodonium salt.

In 2015, Pang et al. developed a Cu-catalyzed one-pot sequential approach for the synthesis of functionalized benzimidazo[1,2-*c*]quinazolines (**32**) from the reaction of *o*-cyanoanilines (**24**) and diaryliodonium salts (**48**) using K_2_CO_3_ as a base (Scheme 66) [123]. The developed method was completed in two steps—initially, the Cu(OTf)_2_-catalyzed synthesis of bromo-substituted quinazolin-4(3*H*)-imines from readily available *o*-cyanoanilines and di-(*o*-bromophenyl)iodonium salt in DCE at 110 °C was performed, and then CuI-catalyzed *N*-arylation delivered the respective fused quinazolines in 93–96% yields.

**Scheme 67:**
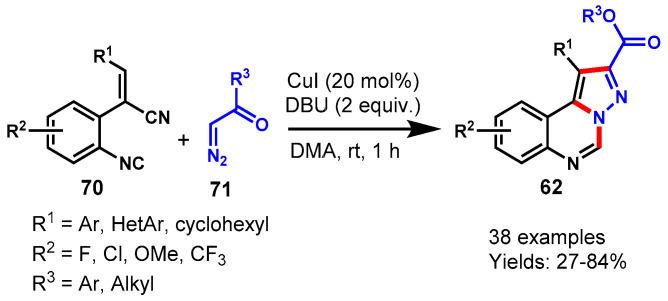
Synthesis of pyrazolo[1,5-*c*]quinazolines from isocyanides and diazo compounds.

In 2020, a CuI-catalyzed tandem reaction between *o*-alkenyl aromatic isocyanides and diazo compounds was demonstrated by the Wang group using DBU as a base in DMF at room temperature (Scheme 67) [124]. A series of isocyanides bearing either electron-donating or electron-withdrawing group at the different positions of the phenyl ring reacted smoothly with diazo compounds and were converted to the corresponding pyrazolo-[1,5-*c*]quinazolines in 27–84% yields. The mechanism of the reaction involved tandem (3 + 2) cyclization, elimination, intramolecular aza-addition sequence in a one-pot manner. The developed approach worked well for the scaled-up synthesis of pyrazolo[1,5-*c*]-quinazolines. The broad substrate scopes, synthetic simplicity, and excellent functional group compatibility are the attractive aspects of this method.

## 7. Nickel-Catalyzed Protocols

Nickel catalysts are highly reactive organometallic species which are less expensive than other members of the group and are used in a variety of organic reactions including cross-dehydrogenative reactions [125], tandem reactions, and cyclization reactions [126]. Furthermore, nickel catalysts have recently been used in the formation of C-C and C-N bonds via hydrogen auto-transfer (HA) and acceptorless dehydrogenative coupling (ADC) reactions [127].

**Scheme 68:**
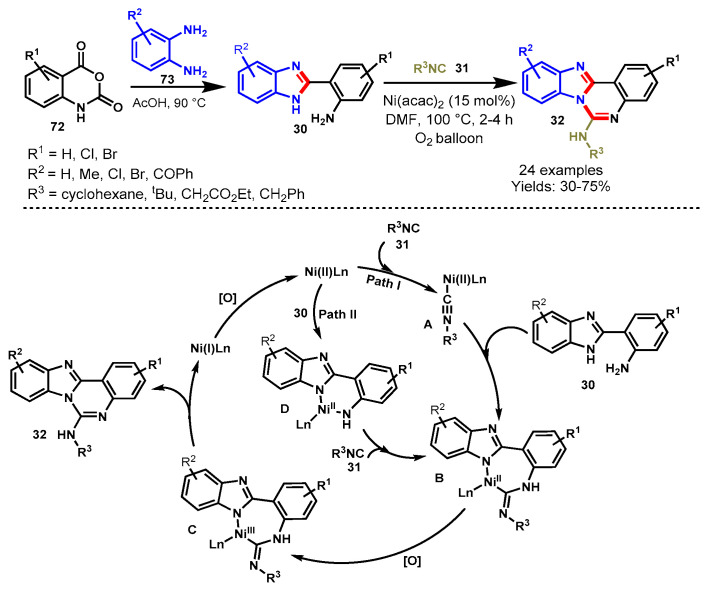
Nickel-catalyzed tandem strategy for the synthesis of quinazolines.

In 2017, Shinde et al. developed a nickel-catalyzed aerobic oxidative isocyanide insertion reaction for the synthesis of quinazolines through a sequential double-annulation cascade approach. Mechanistically, the tandem strategy involved the opening of isatoic anhydride (**72**) and annulation to benzimidazoles (**30**) followed by nickel-catalyzed intramolecular isocyanide (**31**) insertion reactions (Scheme 68) [128]. Various functional group-decorated isatoic anhydrides, ortho-diaminobenzenes (**73**) and isocyanides reacted smoothly under standard reaction conditions to provide the corresponding products in 30–75% yields. The developed protocol successfully applied for the synthesis of naphthalene-fused quinazolines. In addition, the synthesized compounds were also tested for their photophyscial properties. The fluorescence study revealed that electron-withdrawing groups on benzimidazole and a tert-butyl substituent on the amine increased the fluorescence properties of the quinazolines. The salient features of this method were dioxygen as a sole oxidant, base- and ligand-free reaction conditions, the formation of four new C-N bonds in one pot, a short reaction time, and a high bond-forming index (BFI).

**Scheme 69:**
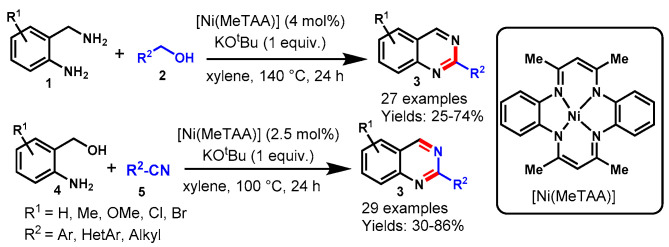
Nickel-catalyzed acceptorless dehydrogenative coupling reaction for the synthesis of quinazolines.

In 2018, Paul and co-workers demonstrated a nickel-catalyzed acceptorless dehydrogenative coupling reaction for the synthesis of quinazolines from the reaction of 2-aminobenzylamines (**1**) with benzyl alcohols (**2**) and 2-aminobenzylalcohols (**4**) with benzonitriles (**5**) in xylene (Scheme 69) [129]. The developed protocol showed a broad range of functional group compatibility on all substrates including ERGs and EWGs to produce the corresponding fused quinazoline products in 25–86% yields. Moreover, to confirm the hydrogen evolution during the quinazoline synthesis, dehydrogenation reactions were performed in the presence of hydrogen acceptors like 4-methoxy-benzaldehyde and the Pd/C-catalyzed hydrogenation reaction of styrene. Notably, the quantification of liberated hydrogen gas was carried out using a gas burette. The broad substrate scope, the Earth-abundant and easy-to-prepare nickel catalyst, and the environmentally benign methodology are the advantageous features of this approach.

**Scheme 70:**
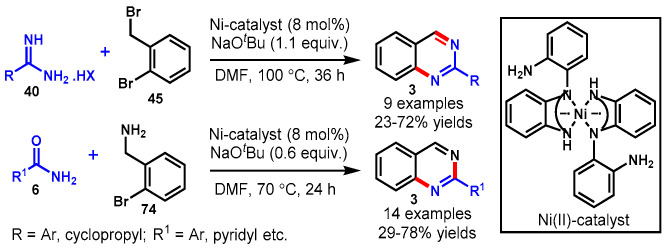
Synthesis of nickel(II)-catalyzed C-2 substituted quinazolines.

In 2019, Paul and co-workers reported the nickel(II)-catalyzed synthesis of quinazolines via the cross-coupling reactions of either benzamide (**6**) and 2-bromobenzylamine (**74**) or amidine (**40**) and 2-bromobenzyl bromide (**45**) in DMF as a solvent under reaction conditions with low variability (Scheme 70) [130]. A wide range of benzamides/amidines reacted well with 2-bromo-benzylamines/2-bromobenzyl bromides under optimized sets of reaction conditions to deliver the desired 2-substituted quinazolines in moderate to good yields. The pyridyl amides and cyclopropyl-substituted amidines could produce the desired products in relatively low yields (23–38%). The authors mentioned that the singlet diradical Ni(II) catalysts used for this transformation are air-stable, easy to prepare, and cheap as compared to the commonly used palladium-based catalysts.

**Scheme 71:**
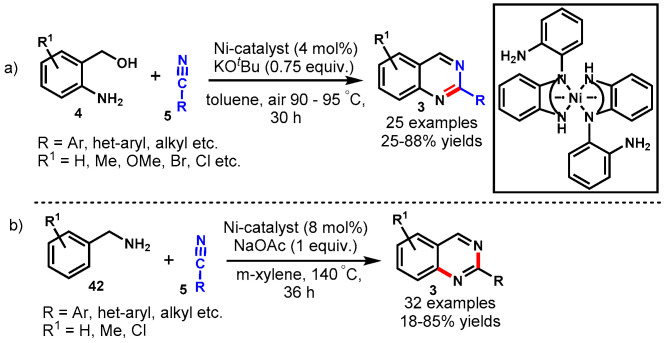
(**a**) Synthesis of C-2 substituted quinazolines from nitriles; (**b**) Nickel-catalyzed annulations of benzyl amine and nitrile.

In 2019, a similar research group further explored the synthesis of quinazolines from the reaction of 2-aminobenzyl alcohols (**4**) and nitriles (**5**) using the previously employed Ni(II) catalyst in toluene at 90 °C (Scheme 71a) [131]. Various aryl/heteroaryl/alkyl nitriles reacted smoothly with 2-aminobenzyl alcohols and yielded the desired quinazolines in moderate to good yields under optimized reaction conditions. Notably, alkyl nitrile, i.e., valeronitrile, produced a relatively lower yield (25%) of the desired product under the same reaction conditions. The reaction proceeded through the formation of 2-aminobenzaldehyde from 2-aminobenzyl alcohol and amides from nitriles. The in situ-generated 2-aminobenzaldehyde and amides underwent base-assisted condensation to produce the corresponding products. Very recently, these authors improved the strategy for quinazoline synthesis through nickel-catalyzed annulations of benzyl amine (**42**) and nitrile (**5**) via C-H activation reactions (Scheme 71b) [132].

## 8. Palladium-Catalyzed Protocols

Palladium-catalyzed cross-coupling reactions have received significant attention in recent years for the development of new methods for heterocycles by means of C-N bond formation. Palladium catalysts are desirable for the synthesis of highly functionalized molecules, medicinally important intermediates, and agro-products due to their wide range of selectivities, simple, efficient, and economical protocols and mild reaction conditions. Therefore, researchers also used palladium as an active catalyst in academia and the pharmaceutical industry for the synthesis of medicinally important quinazoline scaffolds [133].

**Scheme 72:**
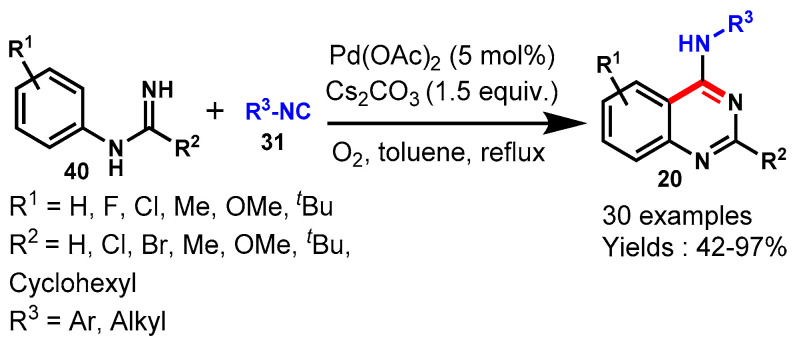
Pd(OAc)_2_-catalyzed synthesis of quinazolines from *N*-arylamidines and isonitriles.

In 2011, Wang et al. developed a Pd(OAc)_2_-catalyzed intramolecular aryl C(sp^2^)-H amidination method for the synthesis of 4-amino-2-aryl(alkyl)quinazolines (**20**) from the reaction of *N*-arylamidines (**40**) and isonitriles (**31**) using Cs_2_CO_3_ as a base in toluene under an oxygen atmosphere (Scheme 72) [134]. Different functional groups decorating *N*-arylamidines like EDGs and EWGs worked efficiently with aliphatic and aromatic isonitrile under optimized conditions to produce the functionalized quinazoline in moderate to excellent yields (42–97%). Notably, in this approach, sterically hindered groups such as *tert*-butylamino and 2,6-dimethylphenylamino were introduced at the C-4 position of the quinazoline ring, which is an important scaffold in medicinal chemistry.

**Scheme 73:**
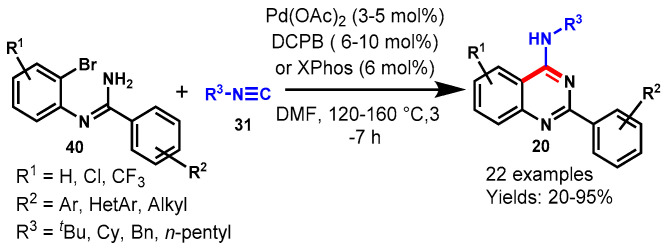
Palladium-catalyzed synthesis of quinazolines from *N*-(2-bromoaryl)amidines and isocyanide.

In 2011, Orru and colleagues demonstrated a Pd(OAc)_2_-catalyzed intramolecular imidoylative cross-coupling reaction in DMF to synthesize 4-aminoquinazolines (**20**) from readily available *N*-(2-bromoaryl)amidines (**40**) and isocyanide (**31**) (Scheme 73) [135]. Diversely substituted amidines reacted well with various aliphatic isocyanides under standard reaction conditions and provided the 4-aminoquinazoline derivatives in moderate to excellent yields (20–95%). However, the developed method did not work with aromatic isocyanides. Furthermore, several synthesized compounds have pharmaceutically important biological activities, such as potent topoisomerase I inhibitors and phosphodiesterase inhibitors, making the developed protocol very useful for future applications in medicinal chemistry.

**Scheme 74:**
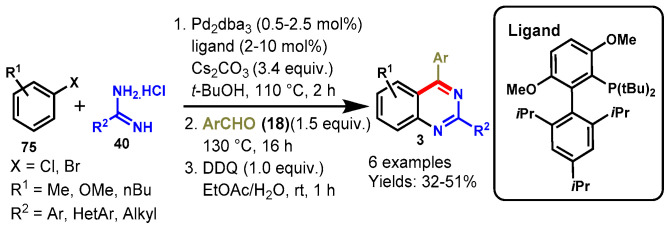
Palladium-catalyzed synthesis of quinazolines from amidines and arylhalides.

In 2012, Buchwald and team explored Pd_2_dba_3_-catalyzed one-pot sequential approach for the synthesis of quinazoline derivatives by reacting amidines (**40**) with arylhalides (**75**) using Cs_2_CO_3_ as base and DDQ as an oxidant (Scheme 74) [136]. Mechanistically, the reaction proceeded through palladium-catalyzed *N*-arylation, base-promoted imine formation followed by electro-cyclization and oxidation. The developed method tolerated a wide range of electron-rich functional groups on arylhalide to provide the desired products in moderate yields. Moreover, heterocyclic amidines and aldehydes also worked well under similar reaction conditions. Unfortunately, EWGs bearing arylhalides failed to produce the desired products.

**Scheme 75:**
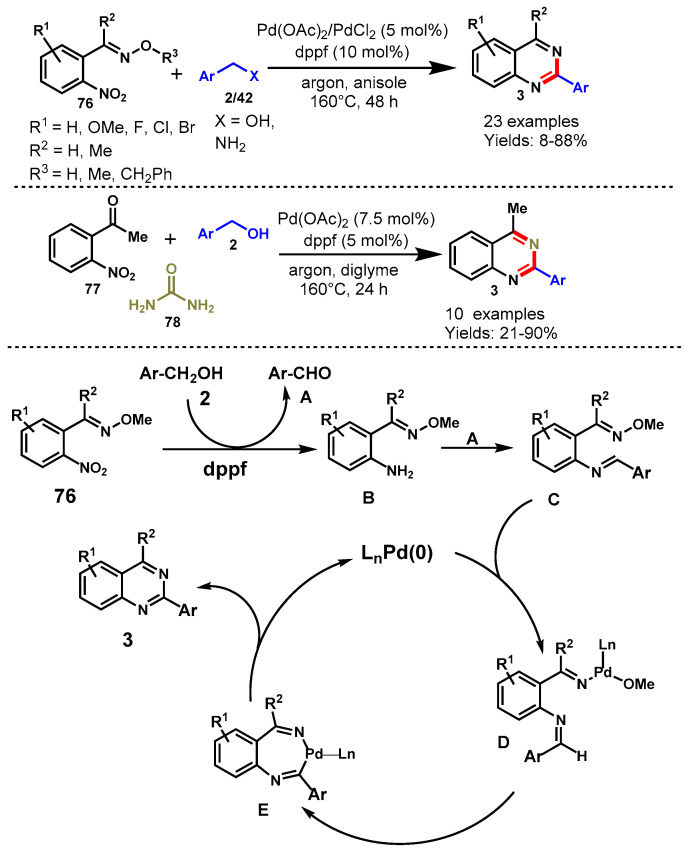
Palladium-catalyzed one-pot synthesis of 2,4-disubstituted quinazolines.

In 2014, a Pd-catalyzed one-pot hydrogen transfer strategy was described by Deng’s group for the synthesis of 2,4-disubstituted quinazolines through the reaction of *E*-1-(2′-nitrophenyl)ethanone *o*-alkyloximes (**76**) with benzyl alcohols (**2**)/benzyl amines (**42**) using dppf as a ligand under an argon atmosphere (Scheme 75) [137]. A variety of benzyl alcohols/benzyl amines bearing ERGs and EWGs groups on the aromatic ring were employed with (*E*)-2-nitrobenzaldehyde *o*-alkyloxime to provide the corresponding quinazoline products in good yields. Heterobenzylic alcohols, i.e., pyridin-3-ylmethanol and furan-2-ylmethanol, also reacted smoothly. Unfortunately, cyclohexanol and 1-butanol could not deliver the corresponding products under similar reaction conditions. Notably, in this approach, the nitro group was reduced in situ by hydrogen generated from the alcohol dehydrogenation step. In addition, the authors also described a one-pot three-component reaction for the synthesis of quinazolines via the reaction of 1-(2-nitrophenyl)ethanone (**77**), urea (**78**) and benzyl alcohols (**2**) under standard reaction conditions, providing the respective products in 21–90% yields.

**Scheme 76:**
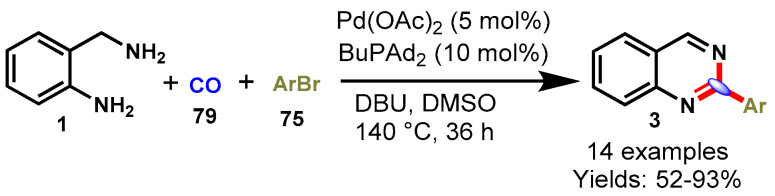
Palladium-catalyzed synthesis of quinazolines from 2-aminobenzylamine and aryl bromides.

In 2014, Wu’s group described a novel and efficient Pd(OAc)_2_-catalyzed carbonylative coupling reaction of 2-aminobenzylamine (**1**) with aryl bromides (**75**) using DBU as the base in DMSO (Scheme 76) [138]. Aryl bromides decorated with various functional groups, such as ERGs (methoxy, dimethylamino, and *tert*-butyl) and EWGs (cyano, trifluoromethyl), were transformed into the corresponding products under optimal reaction conditions. Notably, naphthyl, biphenyl, aryl ketone, and heteroaryl bromides also successfully produced the quinazoline products under similar reaction conditions. Mechanistically, the reaction involved a palladium-catalyzed aminocarbonylation condensation-oxidation sequence in a one-pot one-step manner. Furthermore, DMSO plays important roles as both a solvent and an oxidant in this approach.

**Scheme 77:**
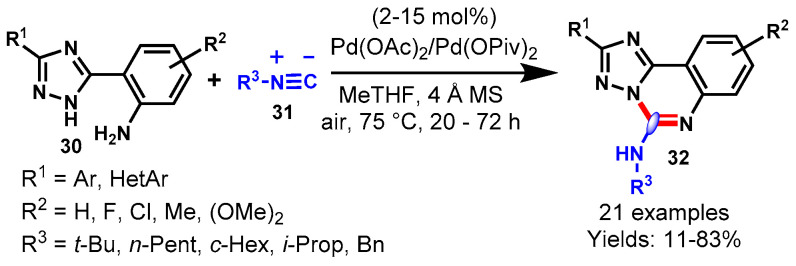
Pd-catalyzed synthesis of quinazolines from (2 aminophenyl)azoles with isocyanides.

In 2014, Ruijter and colleagues described Pd(II)-catalyzed aerobic oxidative coupling of (2-amino-phenyl)azoles (**30**) with isocyanides (**31**) for the synthesis of medicinally important azole-fused quinazolines by using air as an oxidant in MeTHF as a solvent (Scheme 77) [139]. A wide range of triazole derivatives reacted smoothly with isocyanides to provide an annulated product in moderate to excellent yields. Moreover, several hetero-aromatic groups on triazole were also applied successfully for the annulation reaction after the minor tuning of the catalyst loading and reaction time. The developed protocol also worked well for the synthesis of 5-aminotetrazolo[1,5-*c*]-quinazoline and benzimidazoquinazoline under similar reaction conditions.

**Scheme 78:**
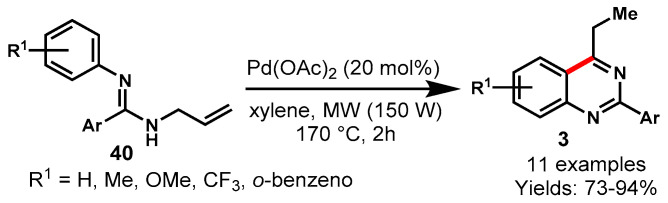
Palladium-catalyzed synthesis of quinazolines from *N*-allylamidines.

In 2015, Wang and his team developed an annulation process for the synthesis of quinazolines from *N*-allyl-amidines (**40**) under microwave irradiation by using palladium as an active catalyst in xylene at 170 °C (Scheme 78) [140]. The scope of this methodology was explored by using a range of aryl amidines to afford the respective product in good to excellent yields (73–94%).

**Scheme 79:**
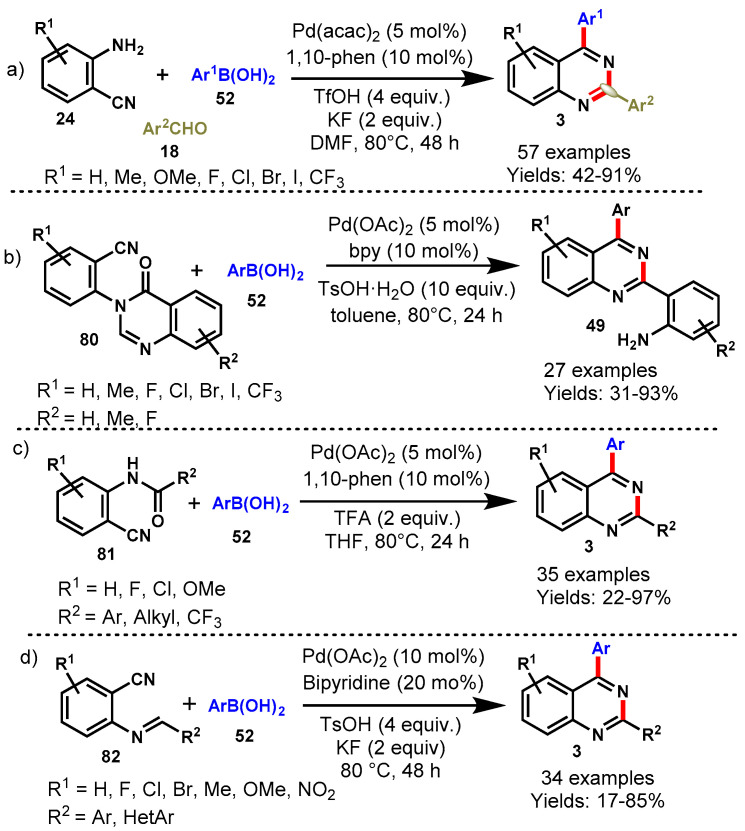
(**a**) Pd-catalyzed synthesis of quinazolines from 2-aminobenzonitriles, aldehydes, and aryl boronic acids; (**b**) Synthesis of quinazolines from 2-(quinazolinone-3(4H)-yl)benzonitriles and aryl boronic acids; (**c**) Synthesis of 2,4-disubstituted quinazolines from aryl boronic acids and N-(2-cyanoaryl)-benzamides; (**d**) Pd-catalyzed tandem addition/cyclization of 2-(benzylidene-amino)benzonitriles with arylboronic acids.

In 2018, Chen group proposed a palladium-catalyzed one-pot, three-component tandem method for the synthesis of quinazolines by using readily available 2-aminobenzonitriles (**24**), aldehydes (**18**), and aryl boronic acids (**52**) in DMF (Scheme 79a) [141]. A variety of functional groups decorating 2-aminobenzonitriles, aldehydes, and aryl boronic acids were compatible under optimized reaction conditions and delivered substituted quinazolines in good to excellent yields (42–91%). 

Subsequently, in the same year, these authors developed another approach for producing quinazoline derivatives through the reaction of 2-(quinazolinone-3(4*H*)-yl)benzonitriles (**80**) and aryl boronic acids (**52**) in toluene (Scheme 79b) [142]. Mechanistically, the reaction proceeded through sequential nucleophilic addition and intramolecular cyclization followed by ring-opening. Interestingly, in this approach, the authors synthesized a new class of 2-(4-arylquinazolin-2-yl) aniline derivatives which were difficult to synthesize in previous methods. The developed protocol was also applicable at the gram scale, leading to the production of quinazolines in 87% yields. The synthetic utility of this method was explored for different kind of reactions, such as acylation, sulfonylation, and the Buchwald–Hartwig coupling reaction.

In a continuation of their work, Chen’s group explored the synthesis of 2,4-disubstituted quinazolines from the reaction of aryl boronic acids (**52**) with *N*-(2-cyanoaryl)-benzamides (**81**) in THF (Scheme 79c) [143]. A variety of functional groups bearing aryl boronic acids reacted smoothly with *N*-(2-cyanoaryl)benzamides to furnish the respective quinazolines in moderate to excellent yields. Moreover, naphthalen-1-ylboronicacid, naphthalen-2-ylboronic acid, and biphenyl-4-ylboronic acid also reacted well under optimized reaction conditions. However, methyl boronic acid and (*E*)-styryl boronic acid could not produce the corresponding products under similar reaction conditions. Mechanistically, the reaction involved nucleophilic addition and imine formation, followed by intramolecular nucleophilic addition and dehydration, leading to the formation of quinazolines.

In 2019, Chen’s group described another Pd-catalyzed tandem addition/cyclization of 2-(benzylidene-amino)benzonitriles (**82**) with arylboronic acids (**52**) for the synthesis of quinazoline derivatives. The mechanism of the reaction involved sequential nucleophilic addition followed by an intramolecular cyclization, leading to the desired products in 17–85% yields (Scheme 79d) [144].

**Scheme 80:**
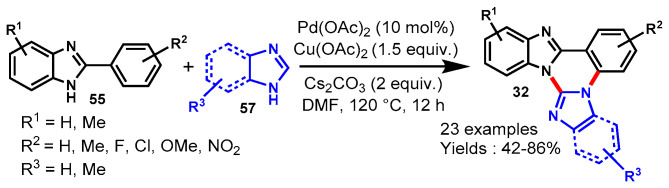
Palladium-catalyzed synthesis of quinazolines from 2-phenyl benzimidazole.

In 2019, Wang and team demonstrated a simple and straightforward method for the synthesis of azole-fused quinazolines (**32**) via a palladium-catalyzed cross-dehydrogenative coupling reaction of 2-phenyl-1*H*-benzo[d]imidazole (**55**) with 1*H*-benzo[*d*]imidazole (**57**) using Cu(OAc)_2_ as the oxidant in DMF (Scheme 80) [145]. Different functional group-containing 2-phenylbenz-imidazole worked well with imidazole/benzimidazole under stabilized reaction condition and delivered the respective fused quinazolines in moderate to excellent yields (42–86%). Moreover, electron-releasing groups like methyl or methoxy on the 2-aryl benzimidazole moiety produced a higher yield than electron-withdrawing groups. Notably, in the case of the NO_2_ functional group, the authors did not observe the target product, which might be due to the reduced electron density on the ring, which disfavors the formation of the palladium chelate.

**Scheme 81:**
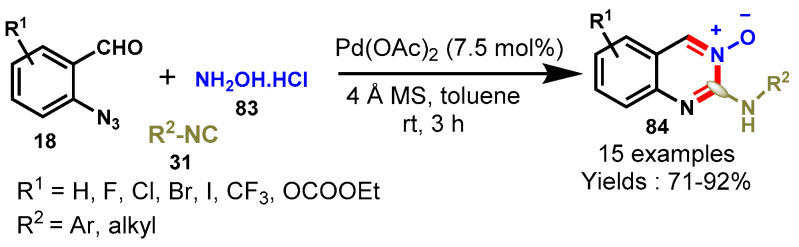
Pd(OAc)_2_-catalyzed synthesis of quinazolines from 2-azidobenzaldehydes.

In 2019, Sawant and team explored a Pd(OAc)_2_-catalyzed one-pot three-component regio-selective strategy for the synthesis of functionalized quinazoline-3-oxides (**84**) via the reaction of 2-azido-benzaldehydes (**18**), isocyanides (**31**) and hydroxylamine hydrochloride (**83**) in toluene at room temperature (Scheme 81) [146]. A range of azidobenzaldehydes and isocyanides reacted well with hydroxylamine hydrochloride under stabilized reaction conditions and delivered the respective quinazolines in good to excellent yields (71–92%). Mechanistically, the reaction proceeded through azide-isocyanide denitrogenative coupling and condensation with hydroxylamine followed by 6-exo-dig cyclization of hydrazones.

**Scheme 82:**
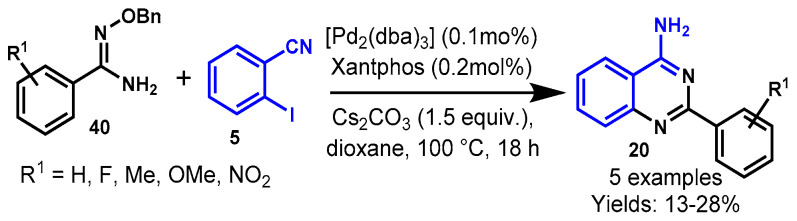
Palladium-catalyzed synthesis of quinazolines from *o*-benzyl benzamide oximes.

In 2019, Demakova et al. described a Pd-catalyzed efficient cascade approach for the synthesis of 4-aminoquinazolines (**20**) by reacting *o*-benzyl benzamide oximes (**40**) with 2-iodobenzonitrile (**5**) using xantphos as the ligand and Cs_2_CO_3_ as the base in dioxane at 100 °C under an argon atmosphere (Scheme 82) [147]. The developed cascade approach involved Pd-catalyzed Buchwald–Hartwig amination and nucleophilic addition followed by protonation and tautomerization, leading to the 4-aminoquinazoline in 13–28% yields. Notably, the developed protocol showed some limitations, such as a smaller substrate scope, a lower yield, and purification difficulties.

## 9. Gold-Catalyzed Protocols

Gold-catalyzed C-H activation, C-H functionalization and cross-coupling reactions have emerged as a potential tool for the development of new methods for heterocyclic compounds. In comparison to other transition metals such as Pt, Pd and Rh, gold has some advantages such as a variable oxidation state (+1 and +3), less toxicity, and the ability to carry out reactions in catalytic amounts without the use of external ligands [148].

**Scheme 83:**
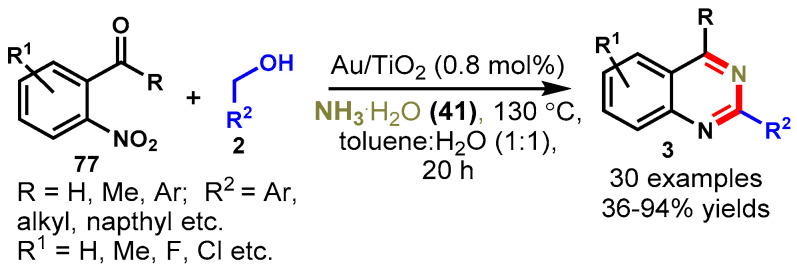
Synthesis of 2,4-disubstituted quinazolines from *o*-nitroacetophenones.

In 2015, Wang and co-workers reported the Au/TiO_2_-catalyzed synthesis of 2,4-disubstituted quinazolines from *o*-nitroacetophenones/*o*-nitrobenzophenones/*o*-nitrobenzaldehyde (**77**) and alcohols (**2**) using aqueous ammonia (**41**) as a nitrogen source via a hydrogen transfer strategy (Scheme 83) [149]. Under optimized conditions, a broad range of benzyl alcohols and aliphatic alcohols underwent the reaction and afforded the corresponding 2,4-disubstituted quinazolines in good to high yields. The steric factor plays a prominent role in product formation. The developed protocol showed good tolerance to the air and moisture and retained the catalytic efficacy of the heterogeneous catalytic system, i.e., gold nanoparticles, which could be reused readily. Moreover, this transformation did not require any additional additive, including an oxidant or reductant.

**Scheme 84:**
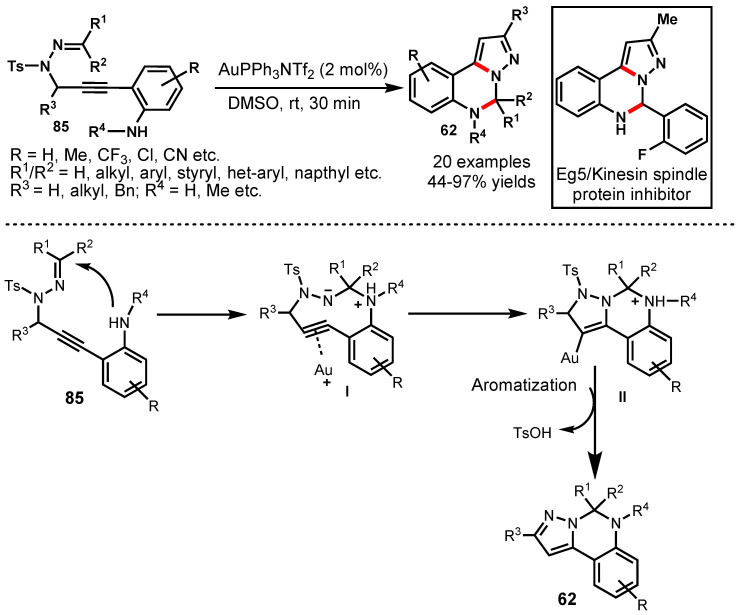
Synthesis of 5,6-dihydropyrazolo[1,5-*c*]-quinazolines.

In 2015, Zhan and his group reported a Au(I)-catalyzed method for the synthesis of 5,6-dihydropyrazolo[1,5-*c*]quinazolines (**62**) via the chemoselective bicyclization of *N*-propargylic sulfonylhydrazones (**85**) in DMSO at room temperature (Scheme 84) [150]. A variety of *N*-propargylic sulfonylhydrazones bearing different functional groups smoothly delivered the corresponding products in good to excellent yields. The developed transformation involved the cyclization of the hydrazone nitrogen instead of the usually favored aniline nitrogen onto the alkyne. The synthetic utility of the present method was explored by synthesizing a potential Eg5/Kinesin spindle protein inhibitor in an 84% yield.

## 10. Ruthenium-Catalyzed Protocols

In recent years, ruthenium-catalyzed C-H activation and annulation reactions have been widely used for the discovery of novel organic chemistry approaches. Ruthenium catalysts are less expensive, stable in both air and water, compatible with oxidants, and work under mild reaction conditions. As a result, ruthenium catalysts have also played an important role in quinazoline synthesis via C-N bond formation reactions [151].

**Scheme 85:**
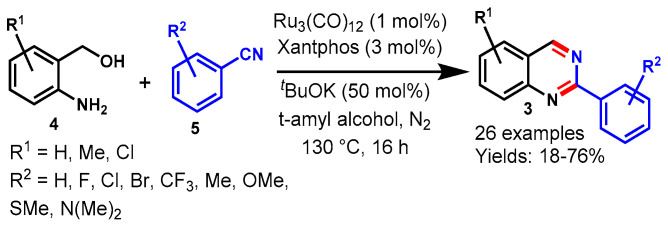
Ru-catalyzed synthesis of quinazolines from 2-aminoarylmethanols and benzonitriles.

In 2014, Jiang and co-workers reported a ruthenium-catalyzed dehydrogenative method for the synthesis of 2-arylquinazolines from the reaction of 2-amino-arylmethanols (**4**) and benzonitriles (**5**) by employing Ru_3_(CO)_12_, xantphos and a *t*-BuOK catalytic system (Scheme 85) [152]. The developed protocol was examined with different substituted 2-aminoaryl-alcohols and benzonitriles under optimized conditions to afford the respective products in 18–76% yields. The method was found to have several advantages such as operational simplicity, broad substrate scope, high atom efficiency, and the use of environmentally benign halogenated reagents.

**Scheme 86:**
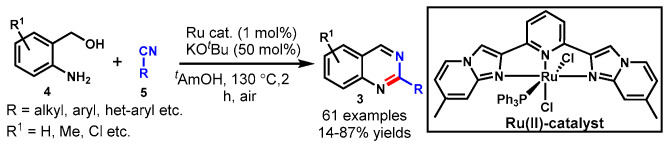
Synthesis of C-2 substituted quinazolines by the dehydrogenative coupling reaction.

In 2019, Wan et al. demonstrated the synthesis of quinazolines via the dehydrogenative coupling reaction of *o*-amino-benzyl alcohols (**4**) and (hetero)aryl or alkyl nitriles (**5**) in the presence of NNN pincer Ru(II)-catalyst (Scheme 86) [153]. The optimized set of conditions successfully produced a variety of C-2 substituted quinazolines in moderate to good yields. Mechanistically, the reaction involved various cascade events such as alcohol oxidation and nitrile hydration followed by cyclo-condensation to yield the respective products. It is noteworthy that both the Ru catalyst and KOtBu are essential to this transformation.

**Scheme 87:**
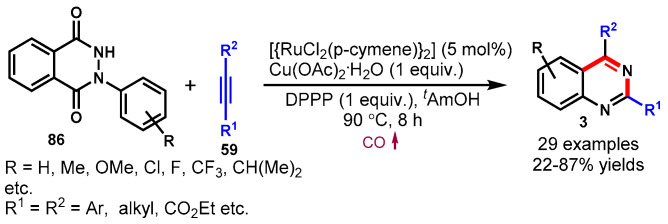
Synthesis of quinazolines from 2-phenyl-dihydrophthalazinediones with alkynes.

In 2018, Gogoi and co-workers reported an unprecedented strategy for the synthesis of 2,4-disubstituted quinazolines from 2-phenyl-dihydrophthalazinediones (**86**) with alkynes (**59**) by applying ruthenium as a catalyst and 1,3-bis(diphenyl-phosphino)propane (DPPP) as a ligand in *tert*-amyl alcohol at 90 °C (Scheme 87) [154]. The reaction pathway comprised a few important steps such as Ru-catalyzed C-H bond activation followed by an annulation reaction through alkyne cleavage. A variety of 2,4-disubstituted quinazolines were successfully synthesized in good to excellent yields under optimized reaction conditions. Notably, the dialkyl-substituted alkynes, terminal alkynes, silyl- and bromo-group-substituted alkynes were not found to be good substrates for this reaction, whereas diaryl-, arylalkyl- and arylester-substituted unsymmetrical alkynes worked well under optimized conditions.

**Scheme 88:**
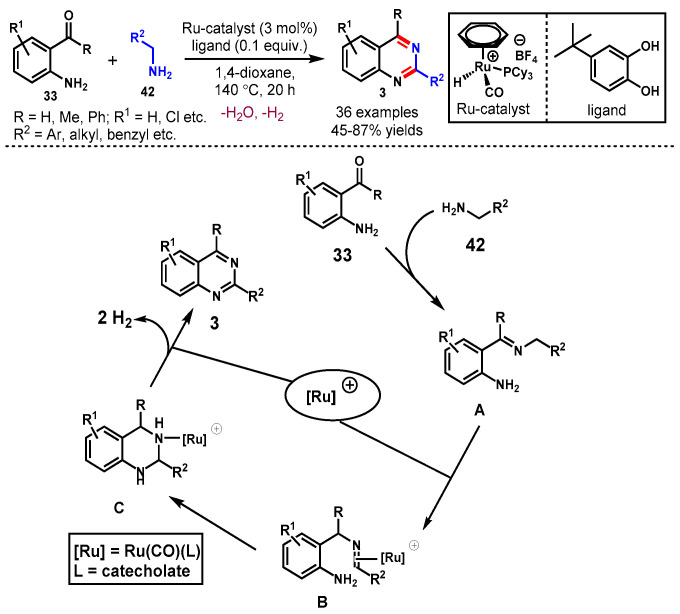
Synthesis of C-2 substituted quinazolines from 2-aminophenyl ketones and amines.

In 2019, Yi and his research group explored the ligand-induced ruthenium-catalyzed synthesis of 2-substituted quinazolines from the coupling reaction of 2-aminophenyl ketones (**33**) with amines (**42**) (Scheme 88) [155]. The in situ-generated ruthenium hydride complex with a catechol ligand was found to exhibit uniquely high catalytic activity and selectivity in forming the quinazolines. The optimized protocol smoothly delivered the respective C-2 substituted quinazolines in moderate to good yields with the elimination of only H_2_O and H_2_ gas as side products. In this process, a variety of benzylamines and alkylamines participated well in the reaction. The developed protocol was successfully extended for the deaminative synthesis of quinazolinones using 2-aminophenyl ketones and benzyl/alkylamides.

## 11. Rhodium-Catalyzed Protocols

Rhodium-catalyzed C-C and C-N bond formation has been rapidly developed in recent years and has become an alternative tool to traditional coupling reactions using organometallic reagents [156,157]. Researchers have successfully synthesized quinazoline analogs using a rhodium catalyst.

**Scheme 89:**
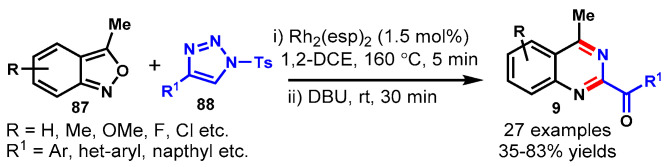
Synthesis of C-2 aroylated quinazolines from *N*-sulfonyl-1,2,3-triazoles and 2,1-benzisoxazoles.

In 2016, Tang and co-workers accomplished an unprecedented Rh(II)-catalyzed trans-annulation of *N*-sulfonyl-1,2,3-triazoles (**88**) with 2,1-benzisoxazoles (**87**) for the synthesis of 2-aroylated quinazolines in DCE solvent (Scheme 89) [158]. A wide range of 4-aryl triazoles reacted smoothly with C-3 methylated 2,1-benzisoxazole bearing several EDGs and EWGs under optimized conditions to provide the desired products in moderate to good yields. The orientation of the substituents on the aryl ring of triazole exerted a notable impact, as the *o*-substituted substrates provided relatively lower yields of the desired products as compared to the m- and p-substituted substrates. Notably, C-3 unsubstituted or 3-phenyl-2,1-benzisoxazoles could not produce the respective products under the developed reaction conditions.

**Scheme 90:**
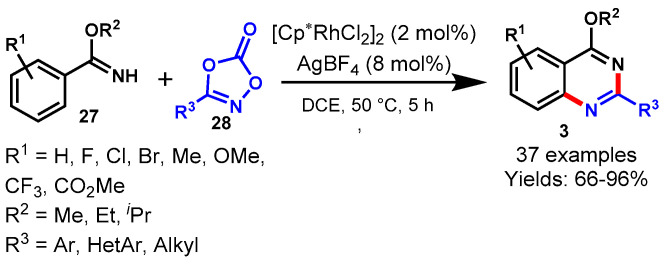
Rh-catalyzed synthesis of quinazolines from benzimidates and dioxazolones.

In 2016, Wang et al. described a Rh(III)-catalyzed double C-N bond formation sequence for the synthesis of substituted quinazolines through intermolecular C-H functionalization of benzimidates (**27**) and dioxazolones (**28**) in DCE (Scheme 90) [159]. Dioxazolone bearing different EDGs and EWGs at different positions of the phenyl ring reacted smoothly with various aryl/heteroarylimidates under standard reaction condition to produce the range of quinazoline derivatives in good to excellent yields (66–96%). Notably, alkyl or heteroaryl substituted dioxazolone derivatives reacted well with arylimidates under similar reaction conditions to afford the corresponding products in 73–87% yields. In addition, dioxazolone played an important role as an internal oxidant to maintain the catalytic cycle of the reactions.

**Scheme 91:**
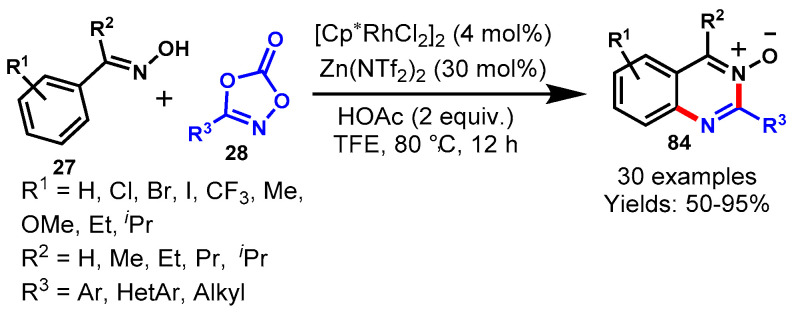
Rh-catalyzed synthesis of quinazolines from ketoximes and dioxazolones.

In 2016, Li and his team developed an efficient tandem approach between ketoximes (**27**) and dioxazolones (**28**) for the synthesis of quinazoline *N*-oxides (**84**) through Rh(III)-catalyzed C-H activation/amidation followed by Zn(II)-catalyzed cyclization/condensation reactions (Scheme 91) [160]. Diversely substituted dioxazolones, including aromatic, heteroaromatic, and aliphatic substituents, reacted smoothly with oximes bearing electron-donating and withdrawing groups on the aryl ring to provide the desired products in moderate to excellent yields. Moreover, other oxime derivatives such as 1-tetralone, propiophenone, and butyrophenone were also successfully participated under similar reaction conditions to afford the corresponding products in good yields. Unfortunately, benzothiophene failed to produce the desired product. The synthetic utility of the developed protocol was demonstrated by the deoxygenation of quinazoline *N*-oxides under Zn, NH_4_Cl, and water reaction conditions.

**Scheme 92:**
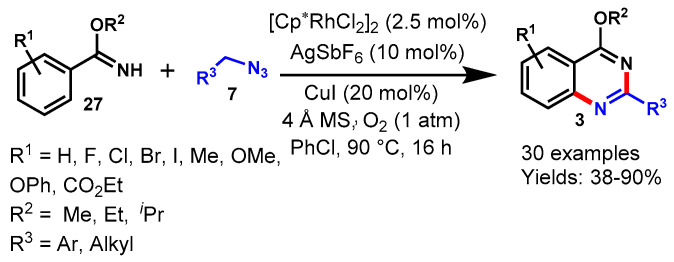
Rh-catalyzed reaction of imidates with alkyl azides for quinazoline synthesis.

In 2016, Jiao and colleagues demonstrated a Rh/Cu co-catalyzed C-H bond activation and annulation approach for the synthesis of quinazolines derivatives through the reaction of imidates (**27**) with alkyl azides (**7**) in chlorobenzene (Scheme 92) [161]. The developed method showed a broad substrate scope and functional group tolerance on both starting materials to provide the respective products in moderate to excellent yields (38–90%). Notably, the simple nonyl azide containing a long carbon chain also worked well under similar reaction conditions and delivered the desired quinazoline in a 38% yield. This method has shown some advantageous features such as good functional group compatibility, high atom efficiency, O_2_ as the terminal oxidant, and azides as a nitrogen source. In addition, this approach provides N_2_ and H_2_O as environmentally benign byproducts.

**Scheme 93:**
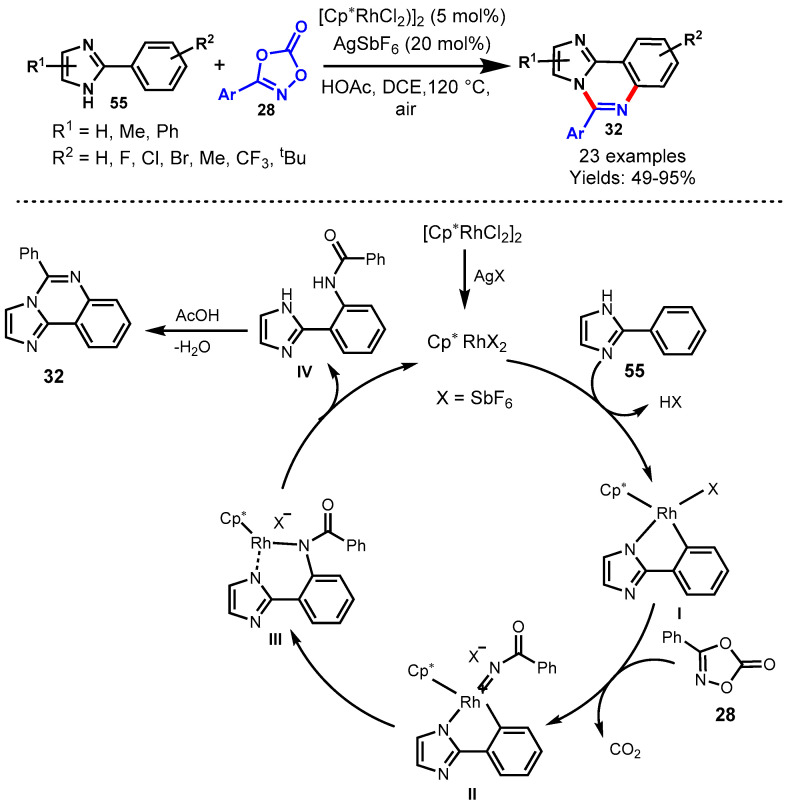
Rhodium-catalyzed synthesis of imidazo [1,2-*c*]quinazolines.

In 2018, Cheng and team described rhodium-catalyzed annulation reaction between 2-arylimidazoles (**55**) and dioxazolones (**28**) for the synthesis of imidazo[1,2-*c*]quinazolines (**32**) in DCE (Scheme 93) [162]. A broad range of 2-arylimidazoles bearing both EDGs and EWGs reacted smoothly with dioxazolone derivatives and successfully delivered the respective fused quinazolines in good to excellent yields (49–95%). However, 2-methylimidazole, 2-ethylimidazole, 2-(piperidin-4-yl)-1*H*-benzo[*d*]imidazole, and 2- benzyl-1*H*-benzimidazole failed to produce the respective products under optimized reaction conditions. Mechanistically, the reaction proceeded through *ortho*-C-H bond amidation followed by intermolecular cyclization. The practicability of this procedure was applicable at a 2 mmol scale, leading to the production of the quinazolines in a 71% yield. The reaction mechanism indicates that intermediate **I** is formed by the chelation-assisted cleavage of ortho C–H bond in the presence of Rh(III) and Ag(I). The intermediate **I** on the reaction with 1,4,2-dioxazol-5-one provides rhodium carbene species **II**, along with the extrusion of CO_2_. The migratory insertion of rhodium carbene species **II** takes place to afford intermediate **III.** Finally, intermediate **III** is converted to **IV** along with Rh(III) species, fulfilling the catalytic cycle. Intermediate **IV** produced quinazoline through intramolecular cyclization via the dehydration reaction.

**Scheme 94:**
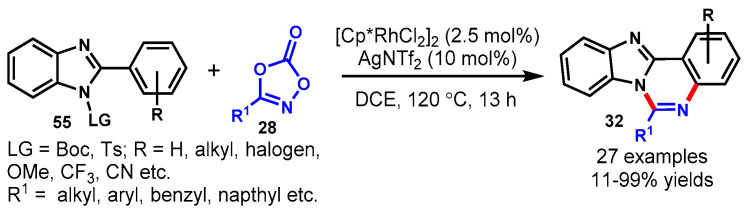
Synthesis of fused quinazolines from amidoxime.

In 2018, Cui and his group reported the synthesis of substituted benzo[4,5]imidazo[1,2-*c*]quinazolines (**32**) through the reaction of *N*-protected-2-phenylbenzoimidazole (**55**) and dioxazolones (**28**) catalyzed by either Rh(III) or Ir(III) catalytic systems (Scheme 94) [163]. The developed protocol showed a broad range of functional group compatibility on both substrates to produce the respective fused quinazoline products in good to excellent yields with only the elimination of CO_2_ as a green by-product. The *o*-methylbenzene- or naphthalene-substituted dioxazolones showed dramatically decreased reactivity, presumably due to the steric hindrance. Notably, the alkyl and benzyl-substituted dioxazolones worked well and produced the desired products in 56–87% yields. The reaction of 2-phenyl-1-tosyl-1*H*-indole with dioxazolone did not produce the respective product under the standard conditions, suggesting that imine was the directing group rather than the quaternary amine.

**Scheme 95:**
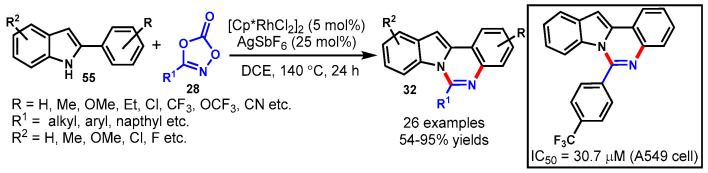
Synthesis of indolo[1,2-c]quinazolines from 2-arylindoles and dioxazolones.

In 2019, Wang et al. reported a Rh(III)-catalyzed C-H amidation and intra-molecular cyclization strategy for the synthesis of indolo[1,2-*c*]quinazolines using 2-arylindole (**55**) and dioxazolone (**28**) substrates in DCE as the solvent (Scheme 95) [164]. The developed method has shown a broad range of substrate and functional group compatibility to produce the desired products in good to excellent yields. The authors highlighted the advantageous features of this method, including the high atom economy, the removal of eco-friendly by-products, i.e., H_2_O and CO_2,_ and the avoidance of any pre-functionalized substrates. The authors also demonstrated the anticancer activity of selected products against PC3, A549, and MCF-7 cancer cell lines. Amongst these, one compound showed considerable activity (IC_50_ = 30.7 µM) against A549 (lung cancer) cell as compared to cisplatin (IC_50_ = 12.2 µM).

**Scheme 96:**
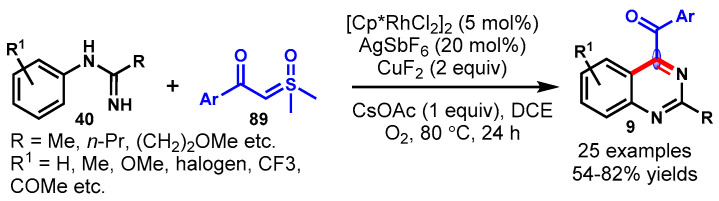
Synthesis of C-4 aroylated quinazolines.

In 2019, Lai et al. explored the Rh-catalyzed synthesis of 4-aroylated quinazolines from the reaction of *N*-arylamidines (**40**) and sulfoxonium ylides (**89**) in the presence of CuF_2_/CsOAc as a combined additive in DCE solvent through C-H bond activation and [5 + 1] annulation steps (Scheme 96) [165]. A broad range of amidines and aryl-substituted sulfoxonium ylides under the developed reaction conditions could provide the desired products in moderate to high yields. However, alkyl-substituted sulfoxonium ylides failed to produce the respective products under optimized conditions. Notably, the use of NaOAc as an additive in the place of CuF_2_/CsOAc successfully led to the formation of 3-aroylated indoles under similar reaction conditions. The selected quinazolines were also tested for their anti-tumor activity. However, none of the tested quinazolines have shown good anti-tumor activity.

**Scheme 97:**
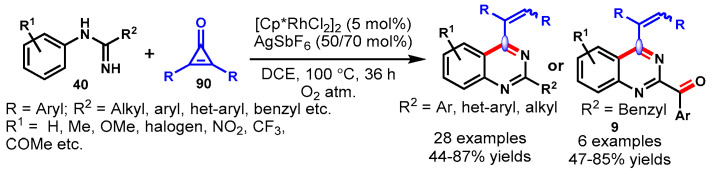
Synthesis of 2-benzoyl-/4-ethenyl quinazolines.

In 2020, Wu and co-workers developed a Rh(III)-catalyzed C-H bond activation followed by [5 + 1] annulation strategy for the synthesis of 4-ethenyl quinazolines from *N*-arylamidines (**40**) with di-arylated cyclopropenones (**90**) in DCM under reflux conditions (Scheme 97) [166]. A variety of 4-ethenyl quinazolines were successfully synthesized under optimized reaction conditions. Importantly, the dialkyl-cyclopropenones and unsymmetrical cyclopropenone were found to be inefficient in the developed reaction conditions. The authors isolated 2-benzoyl quinazolines products when C-benzyl imidamides were treated with cyclopropenones under optimal conditions. This was presumably due to the successive oxidation of benzylic carbon. The broad substrate scopes, high atom economy, and elimination of water as a sole by-product are the appreciable aspects of this method.

**Scheme 98:**
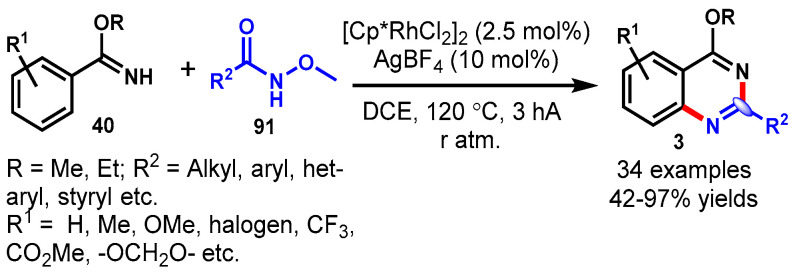
Tandem [4 + 2] annulation approach for the synthesis of quinazolines.

In 2020, for the first time, Dong and co-workers explored a Rh(III)-catalyzed C-H bond activation and tandem [4 + 2] annulation approach for the synthesis of quinazolines through the reaction of *N*-alkoxyamides (**91**) (amide precursor) and benzimidate (**40**) in DCE as a solvent (Scheme 98) [167]. A broad range of aryl imidates and *N*-alkoxyamides (*N*-methoxyarylamides, *N*-methoxy-acetamide, *N*-methoxycinnamamide and *N*-methoxy-heteroarylamides) successfully delivered the desired products in good to excellent yields. The developed approach showed limitations in terms of isopropoxy substituted aryl imidates, which could not produce the respective product under optimal conditions. The authors performed a few synthetic transformations, which included the synthesis of quinazolinone via the acidic hydrolysis of the ethoxy group of quinazoline and directing the group-assisted *o*-CH-amidation of 2-phenylquinazoline.

## 12. Summary and Outlook

In summary, quinazolines and their analogs have taken a leading role in the current research due to the wide variety of their applications in various disciplines of science like medicinal chemistry and material science. This review summarizes an overview regarding the transition metal-assisted synthesis of various quinazolines. Numerous methodologies such as directing group-assisted C-H activation, C-H bond functionalization, condensation followed by intramolecular dehydrative/dehydrogenative cyclizations, cascade reactions, and multi-component processes, etc., have emerged as a powerful tool for the synthesis of quinazolines. Notably, a few of these methods suffer from some limitations, such as a high loading of transition-metal catalysts (equimolar amount), a high temperature, and harsh reaction conditions. Despite the significant development of several fascinating methods, further studies still need to be developed for the synthesis of a wide variety of quinazoline derivatives using sustainable and atom-economic reaction processes under mild and environmentally benign conditions.
